# Reevaluation of the South Asian *MYBPC3*^Δ25bp^ Intronic Deletion in Hypertrophic Cardiomyopathy

**DOI:** 10.1161/CIRCGEN.119.002783

**Published:** 2020-03-12

**Authors:** Andrew R. Harper, Michael Bowman, Jesse B.G. Hayesmoore, Helen Sage, Silvia Salatino, Edward Blair, Carolyn Campbell, Bethany Currie, Anuj Goel, Karen McGuire, Elizabeth Ormondroyd, Kate Sergeant, Adam Waring, Jessica Woodley, Christopher M. Kramer, Stefan Neubauer, HCMR Investigators, Martin Farrall, Hugh Watkins, Kate L. Thomson

**Affiliations:** 1 Radcliffe Department of Medicine, University of Oxford, Oxford, United Kingdom (A.R.H., A.G., E.O., S.N., M.F., H.W., K.L.T.).; 2 Division of Cardiovascular Medicine, John Radcliffe Hospital, Oxford, United Kingdom (A.R.H., A.G., E.O., S.N., M.F., H.W., K.L.T.).; 3 Wellcome Centre for Human Genetics, Oxford, United Kingdom (A.R.H., S.S., A.G., A.W., M.F., H.W., K.L.T.).; 4 Oxford Medical Genetics Laboratories, Churchill Hospital, Oxford, United Kingdom (M.B., J.B.G.H., H.S., C.C., B.C., K.M., K.S., K.L.T.).; 5 Oxford Centre for Genomic Medicine, Oxford University Hospitals NHS Foundation Trust, Oxford, United Kingdom (E.B.).; 6 West Midlands Regional Genetics Laboratory, Birmingham Woman’s and Children’s NHS Foundation Trust, Birmingham, United Kingdom (J.W.).; 7 University of Virginia Health System, Charlottesville, VA (C.M.K.).

**Keywords:** exome, genotype, haplotypes, humans, introns

## Abstract

The common intronic deletion, *MYBPC3*^Δ25^, detected in 4% to 8% of South Asian populations, is reported to be associated with cardiomyopathy, with ≈7-fold increased risk of disease in variant carriers. Here, we examine the contribution of *MYBPC3*^Δ25^ to hypertrophic cardiomyopathy (HCM) in a large patient cohort.

**Methods:**

Sequence data from 2 HCM cohorts (n=5393) was analyzed to determine *MYBPC3*^Δ25^ frequency and co-occurrence of pathogenic variants in HCM genes. Case-control and haplotype analyses were performed to compare variant frequencies and assess disease association. Analyses were also undertaken to investigate the pathogenicity of a candidate variant *MYBPC3* c.1224-52G>A.

**Results:**

Our data suggest that the risk of HCM, previously attributed to *MYBPC3*^Δ25^, can be explained by enrichment of a derived haplotype, *MYBPC3*^Δ25/^^−52^, whereby a small subset of individuals bear both *MYBPC3*^Δ25^ and a rare pathogenic variant, *MYBPC3* c.1224-52G>A. The intronic *MYBPC3* c.1224-52G>A variant, which is not routinely evaluated by gene panel or exome sequencing, was detected in ≈1% of our HCM cohort.

**Conclusions:**

The *MYBPC3* c.1224-52G>A variant explains the disease risk previously associated with *MYBPC3*^Δ25^ in the South Asian population and is one of the most frequent pathogenic variants in HCM in all populations; genotyping services should ensure coverage of this deep intronic mutation. Individuals carrying *MYBPC3*^Δ25^ alone are not at increased risk of HCM, and this variant should not be tested in isolation; this is important for the large majority of the 100 million individuals of South Asian ancestry who carry *MYBPC3*^Δ25^ and would previously have been declared at increased risk of HCM.

## Abstract 2

Supplemental Digital Content is available in the text.

**See Editorial by Sadayappan et al**

Hypertrophic cardiomyopathy (HCM) is the most common inherited cardiac condition, affecting at least ≈1:500 individuals.^1^ It is a genetically heterogeneous disorder, typically attributable to pathogenic variants in genes encoding cardiac sarcomere proteins, predominantly *MYBPC3* and *MYH7*.^2^ Truncating variants in *MYBPC3* are a well-recognized cause of HCM, and the majority are considered to cause autosomal dominant disease with high age-related penetrance; consequently, such variants are extremely rare in the wider nondisease population.^2^

A 25 base pair deletion located within intron 32 of *MYBPC3* (*MYBPC3*^Δ25^), the c.3628-41_3628-17del variant, is a notable exception. Detected in 4% to 8% of individuals of South Asian ancestry,^3,4^ and with an estimated 100 million carriers worldwide, this common variant is considered to be associated with cardiomyopathy, with an almost 7-fold increased risk of cardiomyopathy in heterozygous carriers.^3^ Although previous studies have considered the possibility that *MYBPC3*^Δ25^ lies in linkage disequilibrium with another *MYBPC3* variant that causes or contributes to disease risk,^3,4^ comprehensive analyses in large patient cohorts have not been performed.

Here, using genetic data from 2 large HCM cohorts, we present data suggesting that *MYBPC3*^Δ25^ is not a pathogenic risk factor in HCM. Rather, the increased frequency of this variant in South Asian cardiomyopathy cohorts reflects the enrichment of a derived haplotype, which bears both the common *MYBPC3*^Δ25^ variant and a rare pathogenic variant, *MYBPC3* c.1224-52G>A. Additionally, we find that *MYBPC3* c.1224-52G>A—an intronic variant that is not routinely detected on gene panel or exome sequencing—is the single most common pathogenic variant in individuals of South Asian ancestry in our cohort and the second most common in individuals of European ancestry.

## Methods

The complete methods are available in Materials in the Data Supplement. Due to the confidential nature of some of the research materials supporting this publication, not all of the data can be made accessible to other researchers. Please contact the corresponding author for more information. The study was approved by the local ethics committees, and all patients signed an informed consent.

## Results

### Oxford Medical Genetics Laboratory Demographic and Clinical Details

Within the Oxford Medical Genetics Laboratory (OMGL) cohort, demographic information was available for 98.0% of individuals (2703/2757). The majority of referrals were provided by inherited cardiac condition centers within the United Kingdom (80.1%; 2166/2757). The average age was 54.5 years (±16.2), and 68.4% were men (n=1845; Table 1). No self-identified, or genetically derived, ancestry information was available.

**Table 1.**
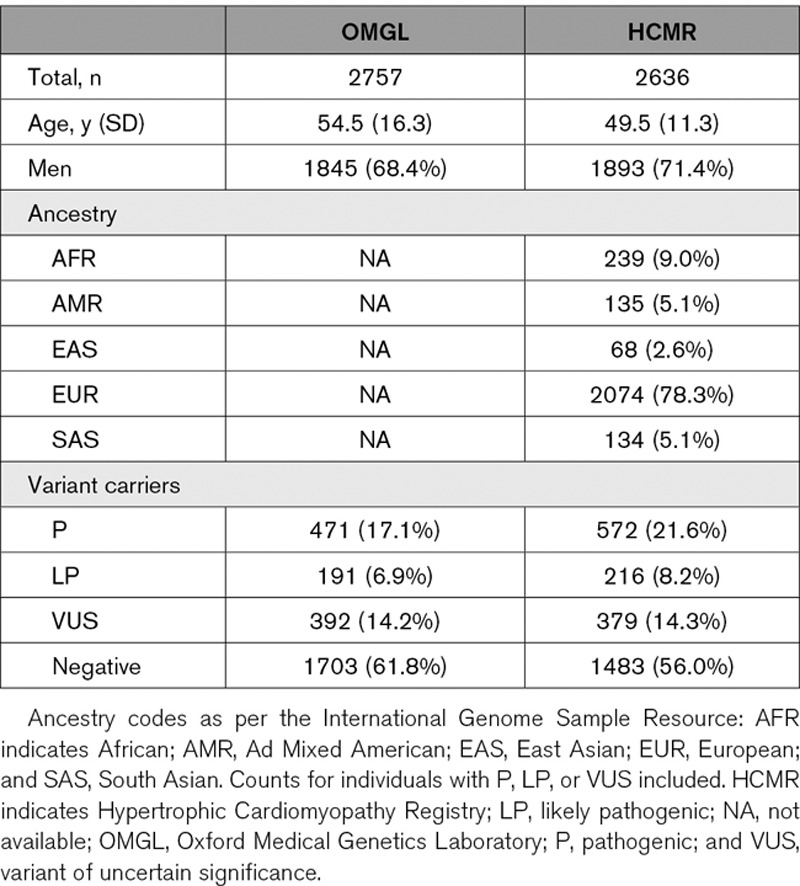
Demographic Summary for OMGL and HCMR Cohorts

### HCMR Demographic and Clinical Details

Within the HCMR cohort, the average age was 49.5 years (±11.3), and 71.4% were men. Genetically derived ancestry predictions, determined through principal components analysis, demonstrated European ancestry in 78.3%, African ancestry in 9.0%, and South Asian ancestry in 5.1% of individuals (Table 1).

### Population Frequency of *MYBPC3*^Δ25^

In the Genome Aggregation Database (gnomAD; v2.1.1), 6.2% of individuals ascribed South Asian ancestry were heterozygous for the *MYBPC3*^Δ25^ variant (943/15 296 [95% CI, 5.7%–6.5%]), 0.1% were homozygous (19). This is consistent with previous studies that have reported frequencies ranging from 2% to 8%.^3,4^ The *MYBPC3*^Δ*25*^ variant is highly specific to individuals of South Asian ancestry: 98.1% (95% CI, 97.0%–98.9%) of *MYBPC3*^Δ*25*^ variant carriers within gnomAD are derived from a South Asian population (Table 2).

**Table 2.**
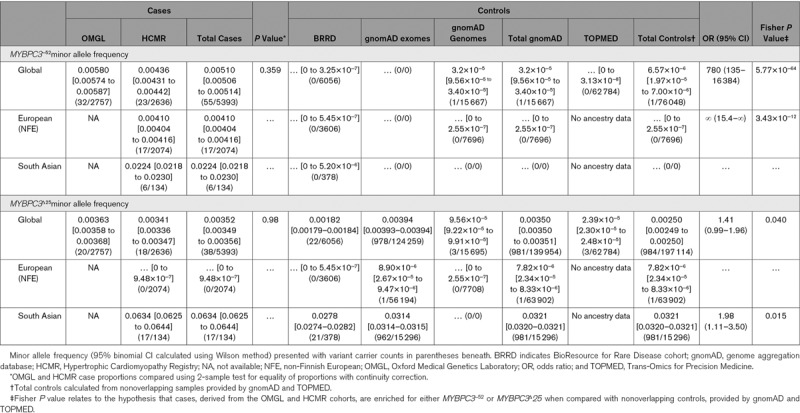
Summary of Allele Frequency Differences Between Cases and Controls

### Oxford Clinical Laboratory Cohort

In the OMGL HCM cohort, pathogenic variants were detected in 17.1% (471/2757), likely pathogenic variants in 6.9% (191/2757), and variants of uncertain significance in an additional 14.2% (392/2757) of individuals. A summary of the most frequently detected variants is presented in Table I in the Data Supplement. 0.7% (20/2757) of individuals were heterozygous for the *MYBPC3*^Δ25^ variant. In 50.0% (10/20) of individuals heterozygous for *MYBPC3*^Δ25^, a pathogenic or likely pathogenic sarcomeric gene variant was also detected; variants of uncertain clinical significance were detected in an additional 3 individuals (15.0%, 3/20; Table 3). Of these accompanying variants, *MYBPC3* c.1224-52G>A was the most frequently observed, found in 30.0% (6/20) of individuals heterozygous for *MYBPC3*^Δ25^.

**Table 3.**
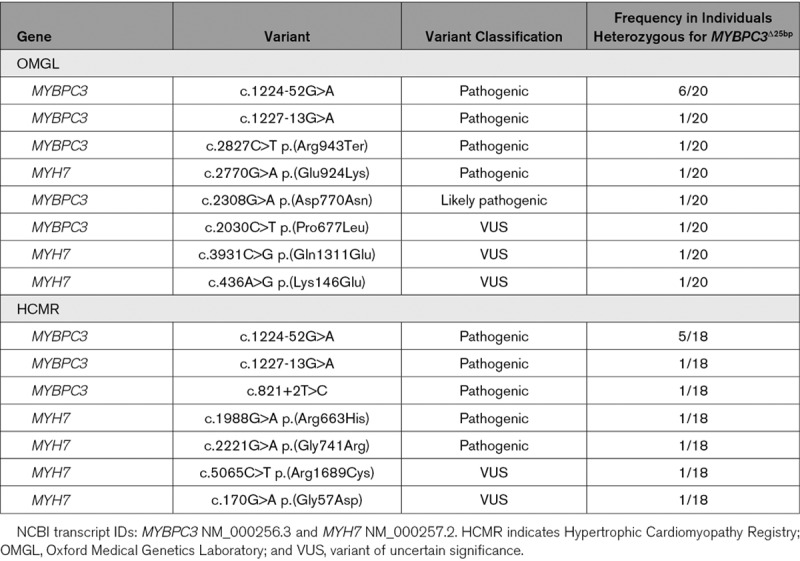
Pathogenic, Likely Pathogenic, and Variants of Uncertain Significance Accompanying ***MYBPC3*** in Individuals From Both the OMGL and HCMR Cohorts

### HCMR Cohort

In the HCMR cohort, pathogenic variants were detected in 21.7% (572/2636), likely pathogenic variants in 8.2% (216/2636), and variants of uncertain significance in an additional 14.4% (379/2636) of individuals. A summary of the most frequently detected variants is presented in Table I in the Data Supplement. Overall, 0.7% (18/2636) of individuals were heterozygous for the *MYBPC3*^Δ25^ variant; no homozygous individuals were detected; 17 *MYBPC3*^Δ25^ variant carriers were ascribed as South Asian ancestry by genetic principal components analysis (94.4%, 17/18). The carrier frequency for *MYBPC3*^Δ25^ within the HCMR South Asian ancestry group was 12.7% ([95% CI, 8.1%–19.4%] 17/134).

In 58.8% (10/17) of South Asian individuals heterozygous for *MYBPC3*^Δ25^, a pathogenic variant in one of the sarcomeric genes was detected (Table 3). An additional 2 individuals were found to have variants of uncertain clinical significance (11.8%, 2/17). Replicating findings from our discovery cohort, the c.1224-52G>A variant was the most frequent, found in 29.4% (5/17) of South Asian individuals heterozygous for *MYBPC3*^Δ25^.

Overall, including the *MYBPC3* c.1224-52G>A variant, 25.4% ([95% CI, 18.8–33.4] 34/134) of HCMR probands ascribed South Asian ancestry had a pathogenic or likely pathogenic sarcomeric gene variant. An additional 15.6% ([95% CI, 10.5–22.8] 21/134) harbored a variant of uncertain significance. This is comparable to the detection rate in the OMGL cohort and to previously published cohorts.^2,5^

Direct comparison of the proportion of heterozygous *MYBPC3*^Δ25^ variant carriers between the HCMR (17/134) and gnomAD (943/15 296) South Asian cohorts indicated a 2-fold enrichment within HCM cases (odds ratio [OR], 2.1 [95% CI, 1.2–3.4]; *P*=0.008). When HCMR probands with the *MYBPC3*^Δ25/^^−52^ haplotype were excluded, no difference was observed (OR, 0.96 [95% CI, 0.40–1.95]; *P*=1.0). Exact multivariate logistic regression, of individuals of South Asian ancestry from the HCMR and BioResource for Rare Disease cohorts (Table 4), provided evidence in support of disease association for the *MYBPC3* c.1224-52G>A variant (OR, 15.90 [95% CI, 2.05–∞]; *P*=0.003) but not the *MYBPC3*^Δ25^ variant (OR, 1.76 [95% CI, 0.77–4.36]; *P*=0.15). The significance of the *MYBPC3* c.1224-52G>A association adjusted for the *MYBPC3*^Δ25^ variant was confirmed using an exact Mantel-Haenszel test (*P*=0.003).

**Table 4.**
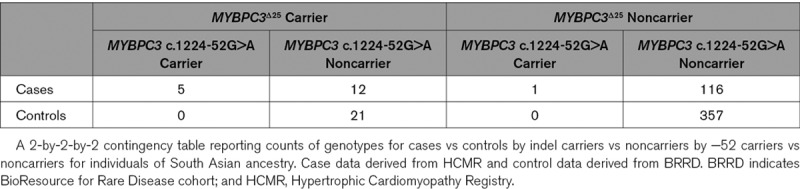
South Asian Cases vs Controls

**Figure 1.**
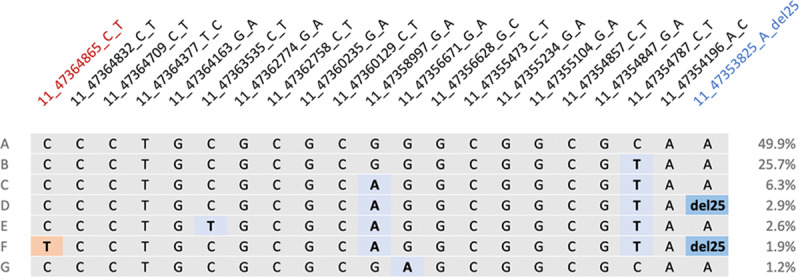
**Haplotype structure across *MYBPC3*.** Each horizontal line (denoted A–G) represents a unique haplotype observed across *MYBPC3* with the South Asian population derived from the Hypertrophic Cardiomyopathy Registry cohort (n=134). Genetic markers denoted using the following nomenclature: <chromosome>_<GRCh37 position>_<reference allele>_<alternate allele>. Grey indicates the presence of the ancestral allele. Blue shading indicates the presence of an alternate allele. The *MYBPC3*^*Δ25*^ allele (11_47353825_A_del25) is emphasized using a darker shade of blue. Red shading represents the presence of the *MYBPC3*^−*52*^ allele (11_47364865_C_T). Haplotype A is composed entirely of reference alleles and is present in 49.9% of the cohort. *MYBPC3*^*Δ25*^ is present on haplotypes D and F. Haplotype F also includes *MYBPC*^−*52*^. Figure generated from data provided by Haploview.

In individuals of South Asian ancestry in the HCMR cohort, the *MYBPC3* c.1224-52G>A variant was found to occur on the second most commonly observed *MYBPC3*^Δ25^ haplotype (Figure 1). Hence, there is evidence of strong linkage disequilibrium between *MYBPC3*^Δ25^ and *MYBPC3* c.1224-52G>A (D′=0.81 and r^2^=0.22; Figure I in the Data Supplement; Table II in the Data Supplement). In South Asian individuals, the *MYBPC3* c.1224-52G>A variant also occurred on a haplotype that did not include the *MYBPC3*^Δ25^ variant.

### Investigating the Pathogenicity of *MYBPC3* c.1224-52G>A

The *MYBPC3* c.1224-52G>A variant (Chr11[GRCh37]:g.47364865C>T, NM_000256.3) was detected in 32 of 2757 (1.2% [95% CI, 0.8%–1.6%]) probands in the OMGL cohort and in 23 of 2636 (0.9% [95% CI, 0.6%–1.2%]) probands in the HCMR cohort. A 2-sample test for equality of proportions, with continuity correction, suggests the minor allele frequencies derived from OMGL and HCMR are equivalent (*P*=0.98). No other pathogenic or likely pathogenic sarcomere gene variants were detected in these cases. Within the OMGL cohort, *MYBPC3* c.1224-52G>A was confirmed to cosegregate with HCM in 4 families (Figure II in the Data Supplement); in 3, it was detected in the proband and 2 other affected relatives. Within the wider HCMR and OMGL populations, *MYBPC3* c.1224-52G>A was found to occur on 2 additional haplotypes, distinct from the 2 South Asian haplotypes, which argues against a unique founder mutation.

The c.1224-52G>A variant occurs once within 76 048 nonoverlapping individuals, present within gnomAD (v.2.1.1) and NHLBI Trans-Omics for Precision Medicine (https://bravo.sph.umich.edu/freeze5/hg38/), indicating a global minor allele frequency, incorporating all available ancestral groups, of 6.57×10^−6^. A comparison of the proportion of individuals heterozygous for this variant in the combined OMGL and HCMR cohorts (55/5393), against these reference populations, generates an extreme effect size (OR, 780 [95% CI, 135–16 384]; *P*=9.74×10^−64^).

**Figure 2.**
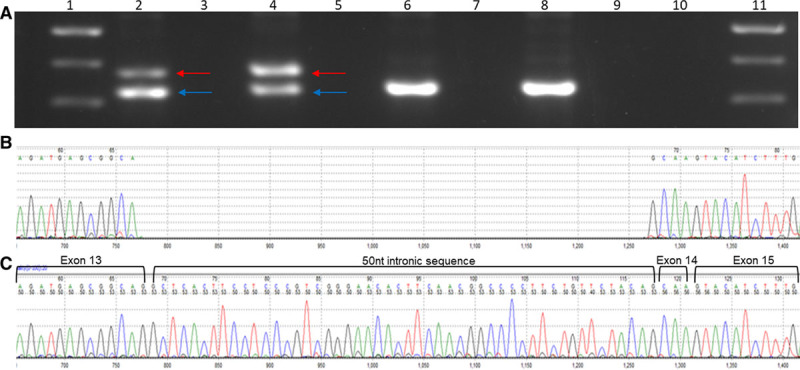
**RNA studies *MYBPC3* c.1224-52G>A variant.** **A**, Gel fractionation of RT-PCR products of lymphocyte-derived RNA from 2 affected individuals heterozygous for the *MYBPC3* c.1224-52A>G. Affected individuals in lanes 2 and 4 (corresponding reverse transcriptase negative controls in lanes 3 and 5) and controls in lanes 6 and 8 (corresponding reverse transcriptase negative controls in lanes 7 and 9). Blue arrow corresponds with normal fragment (323 bp), as seen in controls, and the red arrow corresponds to the aberrant fragment (375 bp). A 100 base pair ladder was used in lanes 1 and 11 (500 bp [dense band], 400 bp, and 300 bp bands shown). **B** and **C**, Sanger sequencing of wild-type (**B**) and aberrant polymerase chain reaction product derived from cDNA of an affected individual harboring *MYBPC3* c.1224-52A>G (**C**) indicates a 50-nucleotide intronic inclusion, confirming in silico splice site predictions.

In silico splice site tools predict that c.1224-52G>A introduces a cryptic splice acceptor site in intron 13 (NM_000256.3), 50 nucleotides upstream (5′) of the native site. Polymerase chain reaction of cDNA reverse transcribed from RNA from 2 individuals with the c.1224-52G>A variant generated an aberrant product. Sequencing of this product confirmed in silico predictions and showed inclusion of 50 intronic nucleotides in the transcript (Figure 2). Inclusion of these nucleotides is predicted to lead to a frameshift in the amino acid sequence and insertion of a premature termination codon at position 438 (p.Ser408fs*31).

### Pathogenicity Classification for *MYBPC3* c.1224-52G>A

Using the American College of Medical Genetics framework,^6^ the *MYBPC3* c.1224-52G>A variant was classified as pathogenic based on the following criteria: PS3: RNA studies have provided evidence of an aberrant effect on splicing (our analyses and published data^7^); PS4: the variant is significantly more frequent in probands with HCM than in population controls; PM2: the variant is very rare in the wider population; and PP1: there is evidence of cosegregation with HCM in multiple families (4 in our cohort and published data^7^).

## Discussion

**Figure 3.**
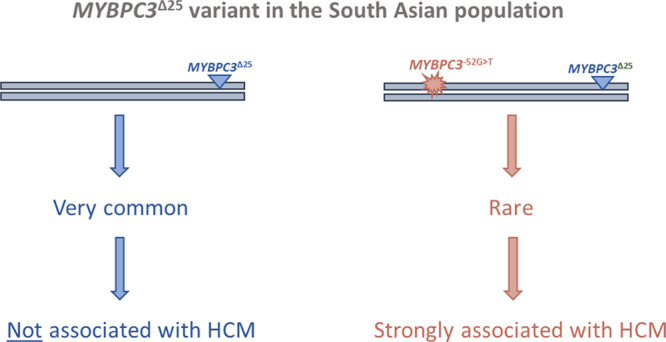
**A reevaluation of the common *MYBPC3*Δ25bp intronic variant (*MYBPC3*^*Δ25*^) in the South Asian population.** The *MYBPC3*^Δ25^ is a common variant present in 4% to 8% of the South Asian population (estimated to be carried by ≈100 million people). In a cohort of South Asian hypertrophic cardiomyopathy (HCM) cases, we detected a rare derived haplotype, bearing both *MYBPC3*^Δ25^ and a pathogenic variant, *MYBPC3* c.1224-52G>A. The rare *MYBPC3*^Δ25/−52^ haplotype is strongly associated with HCM with high penetrance. Haplotypes bearing *MYBPC3*^Δ25^ without the *MYBPC3* c.1224-52G>A variant, which account for the vast majority of South Asian individuals carrying the *MYBPC3*^Δ25^ variant, are not associated with HCM.

When the *MYBPC3*^Δ25^ variant was first reported to be associated with cardiomyopathy in the South Asian population, it was thought likely to have a direct role in disease pathogenesis; since the initial report, it has come to be considered as one of the most compelling examples of a common, low-penetrance variant contributing to the genetic architecture of HCM.^3,8–12^ Genetic analyses undertaken in this study challenge these previous assertions and show that the *MYBPC3*^Δ25^ variant does not directly confer an increased risk of cardiomyopathy but instead acts as a proxy marker for a rare, large effect size, intronic pathogenic variant, *MYBPC3* c.1224-52G>A (Figure 3). Consequently, we conclude that heterozygosity for the *MYBPC3*^Δ25^ common variant is not pathogenic for HCM.

Through RNA studies and segregation analyses, we provide robust evidence to support the pathogenicity of the *MYBPC3* c.1224-52G>A variant. This variant has previously been described in the literature as a pathogenic variant^7^; however, neither its high prevalence nor its relationship with *MYBPC3*^Δ25^ has been reported. Our analyses reveal *MYBPC3* c.1224-52G>A to be a recurrent variant, and one of the most frequent pathogenic variants across all known HCM genes in both European and South Asian populations, comparable to other well-established recurrent and founder pathogenic variants (eg, *MYBPC3* c.2373dup^13^ and *MYBPC3* p.Glu258Lys^2^), and exceeded only by the *MYBPC3* p.Arg502Trp variant, the most common pathogenic variant in HCM.^2,5,14^ Further, the *MYBPC3* c.1224-52G>A variant has a strikingly high OR for disease (≈700), suggesting that it is a high penetrance allele.

Haplotype analyses indicate that an ancestral *MYBPC3* c.1224-52G>A variant arose on a haplotype bearing the common *MYBPC3*^Δ25^ variant and that the reported association between *MYBPC3*^Δ25^ and HCM in the South Asian population was due to the increased frequency of the derived *MYBPC3*^Δ25/^^−52^ haplotype, which had not previously been differentiated from the common *MYBPC3*^Δ25^ haplotype. In our cohort, after accounting for the *MYBPC3*^Δ25/^^−52^ haplotype, the frequency of the *MYBPC3*^Δ25^ allele appears equivalent between HCM cases and reference controls, which casts doubt upon previous pathogenic inferences from risk associations and suggests that it is not clinically appropriate to type the *MYBPC3*^Δ25^ in isolation. Indeed, the ability to detect the *MYBPC3*^Δ25/^^−52^ haplotype is critical not only for individuals with a clinical diagnosis of HCM but for the vast majority of the 100 million individuals of South Asian ancestry heterozygous for the *MYBPC3*^Δ25^ alone, who would previously have been declared at increased risk of HCM.

### Limitations

Our conclusions rely on the observed *MYBPC3*^Δ25^ and *MYBPC3*^Δ25/^^−52^ haplotype frequencies being representative of the wider South Asian population. Here, direct evaluation of *MYBPC3*^Δ25^ and *MYBPC3*^Δ25/^^−52^ and HCM disease risk has relied on analysis performed using individuals ascribed South Asian ancestry based on genetic principal components analysis from 2 independent, but relatively small, cohorts. Large reference cohorts, specifically gnomAD and Trans-Omics for Precision Medicine, were useful in quantifying the allele frequencies of both *MYBPC3*^Δ25^ and *MYBPC3* c.1224-52G>A but were not suitable for the direct evaluation of the *MYBPC3*^Δ25/^^−52^ haplotype, given the lack of individual-level data.

Our case series comprised 2 large HCM cohorts with a combined total of 5394 HCM probands (OMGL, n=2757; HCMR, n=2636), representing the largest published HCM cohort to date. *MYBPC3*^Δ25^ and *MYBPC3*^Δ25/^^−52^ haplotype frequencies were equivalent within these mixed ancestry HCM cohorts. Ancestry data were only available from the HCMR cohort, in which 134 cases were defined as South Asian; additional analyses in other South Asian cohorts will refine *MYBPC3*^Δ25/^^−52^ haplotype frequency estimates and allow more accurate quantification of the strength of the association of this haplotype to HCM in this population.

The findings in this study relate specifically to HCM. In the original case-control study by Dhandapany et al,^3^ 2 composite case groups were assembled that included individuals diagnosed with HCM (n=357), dilated cardiomyopathy (n=395), and restrictive cardiomyopathy (n=15). While our findings refute a pathogenic role for the *MYBPC3*^Δ25^ variant in HCM, at present, our conclusions do not extend to these other cardiomyopathies or to homozygosity for this variant. However, given current understanding of the diametrically opposing molecular mechanisms that underpin sarcomeric HCM and dilated cardiomyopathy,^15–17^ it seems unlikely that a single variant, such as *MYBPC3*^Δ25^, could cause both conditions. Further, truncating variants in *MYBPC3* have only been associated with HCM and not primary dilated cardiomyopathy.^2^

### Conclusions

The results of this study provide strong evidence to refute a direct pathogenic link between the *MYBPC3*^Δ25^ variant and HCM risk; this is important for the very large number of South Asian individuals who will be found to have this variant when undergoing either targeted or genome-wide genetic analysis. Additionally, they highlight *MYBPC3* c.1224-52G>A as an important HCM variant. They also reiterate the importance of sequencing deeper intronic regions in the *MYBPC3* gene, and, indeed, other cardiomyopathy genes where truncating variants are believed to cause the disease. Collectively, these findings have significant implications for our understanding of the genetic architecture of HCM and for the clinical management of patients with HCM.

## Acknowledgments

We thank the National Institute for Health Research (NIHR) BioResource volunteers for their participation and gratefully acknowledge NIHR BioResource centers, National Health Service (NHS) Trusts, and staff for their contribution. We thank the National Institute for Health Research and NHS Blood and Transplant. The views expressed are those of the author(s) and not necessarily those of the NHS, the NIHR, or the Department of Health and Social Care.

## Sources of Funding

A.R. Harper has received support from the Medical Research Council Doctoral Training Partnership. A. Goel has received support from the British Heart Foundation (BHF), European Commission (LSHM-CT-2007-037273 and HEALTH-F2-2013-601456), and TriPartite Immunometabolism Consortium-NovoNordisk Foundation (NNF15CC0018486). A. Waring has received support from the Wellcome Trust. M. Farrall and Dr Watkins have received support from the Wellcome Trust core award (090532/Z/09/Z) and the BHF Centre of Research Excellence, Oxford (RE/13/1/30181). Dr Watkins has received support from the National Institute for Health Research (NIHR) Oxford Biomedical Research Centre. Drs Kramer, Neubauer, and Watkins received support from a National Heart, Lung, and Blood Institute (grant U01HL117006-01A1). Computation used the Oxford Biomedical Research Computing facility—a joint development between the Wellcome Centre for Human Genetics and the Big Data Institute supported by Health Data Research UK and the NIHR Oxford Biomedical Research Centre. Financial support was provided by the Wellcome Trust Core Award (grant No. 203141/Z/16/Z).

## Disclosures

Dr Watkins is a consultant for Cytokinetics. Dr Kramer is a consultant for Cytokinetics and receives research grants from Biotelemetry and MyoKardia. S.N. is a consultant for Pfizer and Cytokinetics and receives research grants from Boehringer Ingelheim. The other authors report no conflicts.

## Supplementary Material



## Appendix

HCMR Investigators: Theodore Abraham, MD, Hypertrophic Cardiomyopathy Center of Excellence, Johns Hopkins University, Baltimore, MD; Lisa Anderson, MD, St George’s University Hospitals NHS Trust, London, United Kingdom; Evan Appelbaum, MD, Departments of Medicine, Cardiovascular Division & Radiology, Beth Israel Deaconess Medical Center, Harvard Medical School, Boston, MA; Camillo Autore, MD, Division of Cardiology, Department of Clinical & Molecular Medicine, St. Andrea Hospital, Sapienza University, Rome, Italy; Colin Berry, MD, British Heart Foundation Glasgow Cardiovascular Research Center, Institute of Cardiovascular & Medical Sciences, University of Glasgow, UK; Elena Biagini, MD, Cardio-Thoraco-Vascular Department, University Hospital of Bologna, Policlinico S. Orsola-Malpighi, Bologna, Italy; William Bradlow, MD, Department of Cardiology, New Queen Elizabeth Hospital Birmingham, UK; Chiara Bucciarelli-Ducci, MD, Bristol Heart Institute, Bristol National Institute of Health Research (NIHR) Biomedical Research Center, University Hospitals Bristol NHS Trust & University of Bristol, UK; Amedeo Chiribiri, MD, PhD, Cardiovascular Division, King’s College London British Heart Foundation Center of Excellence, The Rayne Institute, St. Thomas’ Hospital Campus, London, UK; Lubna Choudhury, MD, Division of Cardiology, Department of Medicine, Bluhm Cardiovascular Institute, Northwestern University Feinberg School of Medicine, Chicago, IL; Andrew Crean, MD, Division of Cardiology, Peter Munk Cardiac Center, University Health Network, University of Toronto, Ontario, Canada; Dana Dawson, MD, Aberdeen Cardiovascular & Diabetes Center, University of Aberdeen, UK; Milind Y. Desai, MD, Department of Cardiovascular Medicine, Center for Radiation Heart Disease, Heart & Vascular Institute, Cleveland Clinic, Cleveland, OH; Eleanor Elstein, MD, Division of Cardiology, Department of Medicine, Royal Victoria Hospital, McGill University Health Center, Montreal, Quebec, Canada; Andrew Flett, MD, Department of Cardiology, University Hospital Southampton NHS Foundation Trust, Southampton, UK; Matthias Friedrich, MD, Department of Medicine, Heidelberg University, Heidelberg, Germany; Stephen Heitner, MD, Oregon Health & Sciences University (OHSU), Division of Cardiovascular Medicine, Knight Cardiovascular Institute, Portland, OR; Adam Helms, MD, Department of Internal Medicine, University of Michigan, Ann Arbor, MI; Carolyn Ho, MD, Cardiovascular Division, Brigham and Women’s Hospital, Boston, MA; Daniel L. Jacoby, MD, Section of Cardiovascular Medicine, Department of Internal Medicine, Yale School of Medicine, New Haven, CT; Han Kim, MD, Duke Cardiovascular Magnetic Resonance Center & Division of Cardiology, Duke University Medical Center, Durham, NC; Bette Kim, MD, Mount Sinai West, Icahn School of Medicine at Mount Sinai, New York City, NY; Eric Larose, MD, Quebec Heart & Lung Institute, Laval University, Quebec, Canada; Masliza Mahmod, MD, Division of Cardiovascular Medicine, Radcliffe Department of Medicine, University of Oxford, UK; Heiko Mahrholdt, MD, Department of Cardiology, Robert-Bosch-Krankenhaus, Stuttgart, Germany; Martin Maron, MD, Hypertrophic Cardiomyopathy Center & Research Institute, Tufts Medical Center, Boston, MA; Gerry McCann, MD, Department of Cardiovascular Sciences, University of Leicester, UK; Michelle Michaels, MD, Erasmus University, Rotterdam, the Netherlands; Saidi Mohiddin, MD, Barts Heart Center, The Cardiovascular Magnetic Resonance Imaging Unit, St Bartholomew’s Hospital, London, UK; Sherif Nagueh, MD, Methodist DeBakey Heart & Vascular Center, Houston, TX; David Newby, MD, Center for Cardiovascular Science, University of Edinburgh, UK; Iacopo Olivotto, MD, Cardiomyopathy Unit & Genetic Unit, Careggi University Hospital, Florence, Italy; Anjali Owens, MD, Center for Inherited Cardiovascular Disease, Division of Cardiovascular Medicine, Perelman School of Medicine, University of Pennsylvania, Philadelphia, PA; F. Pierre-Mongeon, MD, Montréal Heart Institute, Canada; Sanjay Prasad, MD, National Heart & Lung Institute, Imperial College London & Royal Brompton Hospital, London, UK; Ornella Rimoldi, MD, Vita Salute University & San Raffaele Hospital, Milan, Italy; Michael Salerno, MD, Department of Medicine, University of Virginia, Charlottesville, VA; Jeanette Schulz-Menger, MD, Charité, Medical Faculty of the Humboldt University, Experimental & Clinical Research Center and Helios Clinics, Cardiology, Berlin, Germany; Mark Sherrid, MD, Hypertrophic Cardiomyopathy Program, Leon Charney Division of Cardiology, Department of Medicine, New York University School of Medicine, New York, NY; Peter Swoboda, MD, Department of Cardiovascular Imaging Science, Leeds Institute of Cardiovascular & Metabolic Medicine, University of Leeds, UK; Albert van Rossum, MD, Department of Cardiology, Amsterdam UMC, HZ Amsterdam, the Netherlands; Jonathan Weinsaft, MD, Departments of Medicine & Radiology, Weill Cornell Medical College, New York, NY; James White, MD, Calgary Foothills Medical Center, University of Calgary, Alberta, Canada; Eric Williamson, MD, Department of Radiology, Mayo Clinic, Rochester, MN.
