# Supplementary material for: Reevaluation of the South Asian MYBPC3Δ25bp Intronic Deletion in Hypertrophic Cardiomyopathy
Source: Circ Genom Precis Med. 2020 Mar 12;13(3):e002783. doi: 10.1161/CIRCGEN.119.002783 (PMC7299222; doi:10.1161/CIRCGEN.119.002783)
Supplement: Supplementary file 1 [file hcg-13-e002783-s001.pdf]

SUPPLEMENTAL MATERIAL

METHODS

Oxford clinical laboratory cohort

Analysis of core sarcomeric genes (*MYBPC3*, *MYH7*, *TNNI3*, *TNNT2*, *MYL2*, *MYL3*, *ACTC1*, *TPM1)* was undertaken in 2,757 probands referred by inherited cardiac condition specialists to the Oxford Medical Genetics Laboratory (OMGL) for HCM genetic testing between May 2013 and December 2018. Self-identified ancestry data is not available for this cohort; however, prior analysis of this cohort has suggested >80% of probands are of European ancestry.[1,2] Target DNA sequences were enriched using a custom-designed HaloPlex kit (Agilent) before being sequenced on an Illumina MiSeq instrument. Sequence data were adapter-trimmed using Cutadapt[3]; in addition, 5 bp from each end of the reads were cropped to remove any restriction enzyme footprints, and short as well as low-quality reads were discarded using Trimmomatic[4]. Filtered reads were mapped on the human reference genome (hs37d5 assembly) using BWA-mem.[5] Samples were haplotype called and jointly genotyped with an in-house pipeline adapted from the GATK Best Practices[6], using GATK version 4.0.11.0 and Picard version 2.9.2.[7] All variants were confirmed by Sanger sequencing. Variants were assigned a pathogenicity classification in accordance with published guidelines.[8]

Hypertrophic Cardiomyopathy Registry cohort (HCMR)

Analysis of core sarcomeric genes (*MYBPC3*, *MYH7*, *TNNI3*, *TNNT2*, *MYL2*, *MYL3*, *ACTC1*, *TPM1)* was undertaken in 2,636 probands with a clinical diagnosis of HCM recruited into the HCMR project from 2013 to 2018. Clinical details and study design of the HCMR project have been previously reported.[9] Target DNA sequences were enriched using a custom-designed TruSeq kit (Illumina) before being sequenced on an Illumina MiSeq instrument. Target-specific

primers and Illumina adapters were removed from the sequence data using Cutadapt.[3] Short as well as low-quality reads were discarded using Trimmomatic.[4] Filtered reads were mapped on the human reference genome (hs37d5 assembly) using BWA-mem.[5] Samples were haplotype called and jointly genotyped with an in-house pipeline adapted from the GATK Best Practices[6], using GATK version 4.0.11.0 and Picard version 2.9.2[7]. Variants were visually confirmed through inspection of BAM files. Variants were assigned a pathogenicity classification in accordance with published guidelines.[8] The HCMR cohort underwent genome-wide genotyping using the Axiom Precision Research Array (Affymetrix). Genotypes first underwent linkage disequilibrium pruning using PLINK (--indep-pairwise 1000 50 0.05), before FlashPCA2[10] was used to project ancestry-informative principal components present within the 1000 Genomes phase 3 cohort onto the HCMR cohort[11]. A multinomial logistic regression model classified ancestral groups as per the International Genome Sample Resource (http://www.internationalgenome.org/category/population/).

Reference control data

Reference control genotype data for case-control analyses was obtained from the Genome Aggregation Database (gnomAD, v2.1.1)[12,13], the NHLBI Trans-Omics for Precision Medicine (TOPMed) Whole Genome Sequencing Program BRAVO variant browser[14,15], and the Bioresource for Rare Disease (BRRD) cohort.[16] Details relating to the BRRD cohort have been previously published.[16]

Statistical analysis

Statistical analyses were undertaken using R version 3.5.3. Continuous normally distributed variables are presented as mean ± standard deviation (SD). Fishers exact test (FET) and odds

ratio (OR) analyses were undertaken to compare the proportion of individuals heterozygous for the *MYBPC3*$^{\Delta 25}$ variant in South Asian individuals from the HCMR cohort and the Genome Aggregation Database (gnomAD, v2.1.1). 95% confidence intervals (CI) were included for effect size estimates. Binomial confidence intervals for variant proportions were calculated using the exact (Clopper-Pearson) method. Data from the HCMR South Asian cohort and the BRRD South Asian cohort was analysed with the R *elrm* function (https://cran.r-project.org/src/contrib/Archive/elrm/), a Monte Carlo Markov Chain algorithm that approximates exact conditional inference for logistic regression models.[17] This method was used to model the independent effects of the *MYBPC3*$^{\Delta 25}$ and *MYBPC3* c.1224-52G>A variants whilst indirectly allowing for linkage disequilibrium between the two variants through their associations with HCM risk. An exact Mantel-Haenszel test was employed, using the R mantelhaen.test function, to test the association between *MYBPC3* c.1224-52G>A and HCM, adjusted for *MYBPC3*$^{\Delta 25}$.

Haplotype analysis

Using gene panel sequence data specific to individuals of South Asian genetic ancestry from the HCMR cohort, haplotype analysis was conducted using Haploview (version 4.2). Genetic markers located within *MYBPC3*, specifically between positions 47353825 and 47364865 (GRCh37), minimum genotyping rate of 90%, Hardy-Weinberg p-value cut-off of 0.001 and a maximum of one Mendel error, were considered alongside *MYBPC3* c.1224-52G>A and *MYBPC3*$^{\Delta 25}$. Additionally, haplotype analysis using short tandem repeats that span ~4.6 Mb around *MYBPC3* was performed using PHASE (v2.1.1) in all individuals of South Asian ancestry from the HCMR cohort, and in all individuals in the HCMR and OMGL cohorts that carried the *MYBPC3*$^{\Delta 25}$ variant and / or the *MYBPC3* c.1224-52G>A variant.

Investigating the pathogenicity of *MYBPC3* c.1224-52G>A

RNA analysis

A combination of *in silico* tools (SpliceSiteFinder-like, MaxEntScan, NNSplice and Human Splicing Finder), designed to predict the impact of variants on 3' and 5' splice sites were consulted through the splicing module of Alamut® Visual 2.7.1. Total RNA was extracted from lymphocytes derived from peripheral blood of two affected individuals with the *MYBPC3* c.1224-52G>A variant and reverse-transcribed to cDNA using standard protocols. PCR was performed using primers targeted at exon 12 and exon 16 of the *MYBPC3* gene and electrophoresed on an agarose gel. PCR amplicons were gel-purified and Sanger sequenced.

			Counts	
Gene	HGVS.c	HGVS.p	OMGL (n=2,757)	HCMR (n=2,636)
MYBPC3	c.1504C>T	p.Arg502Trp	58	39
MYBPC3	c.2373dup	p.Trp792ValfsTer41	11	45
MYBPC3	c.772G>A	p.Glu258Lys	23	40
MYBPC3	c.1224-52G>A	-	32	23
MYBPC3	c.1624+4A>T	-	19	16
MYBPC3	c.1484G>A	p.Arg495Gln	8	17
MYH7	c.2389G>A	p.Ala797Thr	12	16
MYBPC3	c.1624G>C	p.Glu542Gln	15	13
MYBPC3	c.1928-2A>G	-	7	14
MYBPC3	c.3330+2T>G	-	NA	13

Supplementary Table 1. Most frequent pathogenic or likely pathogenic variants seen across OMGL and HCMR cohorts

A

		MYBPC3 c.1224-52G>A		
		**-52/-52**	**-52/+**	**+/+**
**MYBPC3$^{\Delta25}$**	**Del/del**	0	0	0
	**Del/+**	0	5	12
	**+/+**	0	1	116

B

**Haplotypes**	**Haplotype frequencies**	
**Haplotypes MYBPC3$^{\Delta25}$ - MYBPC3 c.1224-52G>A**	**LD model**	**Equilibrium model**
**Del/-52**	0.018	0.001
**+/-52**	0.004	0.021
**Del/+**	0.045	0.062
**+/+**	0.933	0.916

C

	**Predicted haplotype counts**	
	**MYBPC3 c.1224-52G>A**	**+**
**MYBPC3$^{\Delta25}$**	4.8	12.1
**+**	1.1	250.0

Odds ratio measure of "allelic association" = 93.3

Supplementary Table 2. Haplotype analysis. Panel A: Observed occurrences of *MYBPC3* c.1224-52G>A relative to *MYBPC3*$^{\Delta25}$ within the HCMR South Asian cohort (n=134); Panel B: Reported haplotype frequencies under a linkage disequilibrium model; Panel C: Predicted haplotype counts given observed counts and linkage disequilibrium estimates between genetic markers. + indicates the presence of the wild-type (common, assumed ancestral) allele

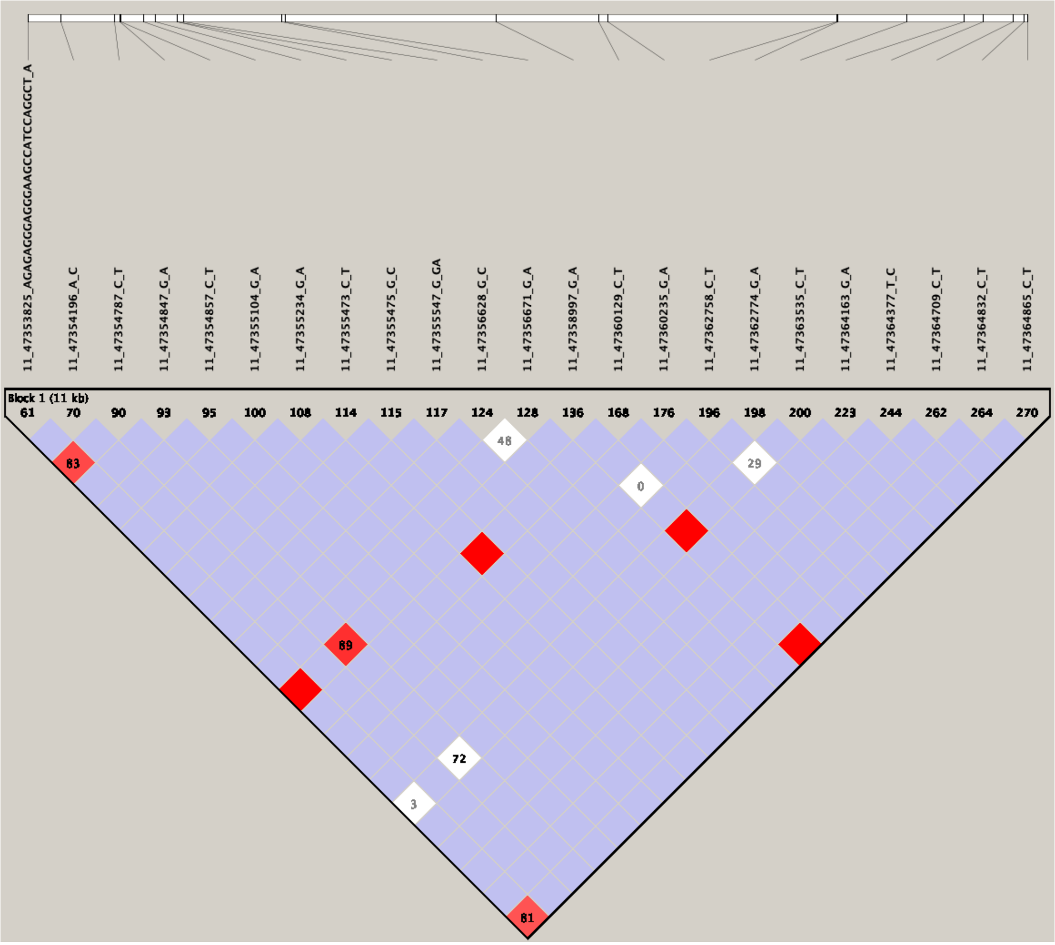

Supplementary Figure 1. Linkage disequilibrium plot for *MYBPC3*. Generated using Haploview, red squares indicate high linkage disequilibrium (LD) between markers. Between marker 61 (MYBPC3$^{\Delta 25}$) and marker 270 (MYBPC3 c.1224-52G>A) there is evidence of high LD (D′ = 0.81 and r^2 = 0.22).

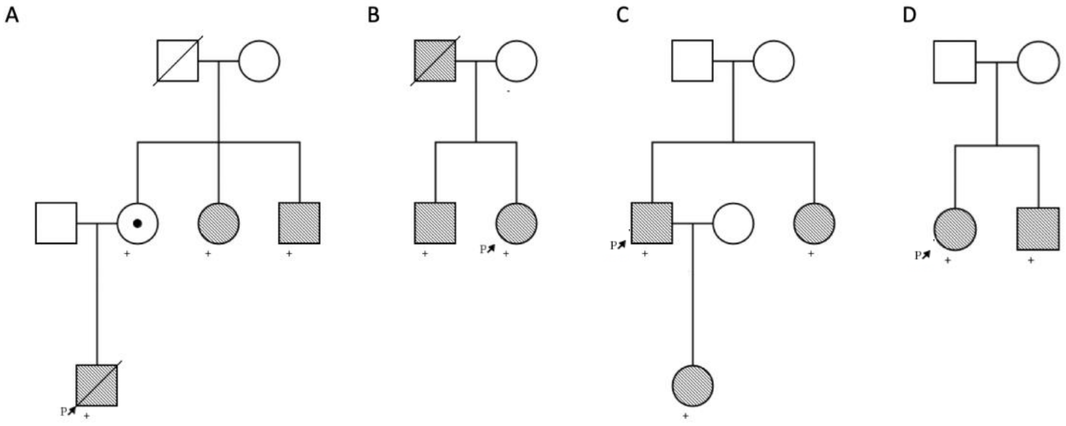

Supplementary Figure 2. Segregation analysis *MYBPC3* c.1224-52G>A variant. Shaded squares represent males with a clinical diagnosis of HCM. Shaded circles represent females with a clinical diagnosis of HCM. White circle with a black dot represent a genotype +ve, phenotype negative female. Letter P and arrow identifies the Proband, defined as the first individual referred for genetic testing. + represents heterozygous for the *MYBPC3* c.1224-52G>A variant.

References

1. Thomson KL, Ormondroyd E, Harper AR, Dent T, McGuire K, Baksi J, Blair E, Brennan P, Buchan R, Bueser T, et al. Analysis of 51 proposed hypertrophic cardiomyopathy genes from genome sequencing data in sarcomere negative cases has negligible diagnostic yield. *Genet Med*. 2019;21(7):1576–1584.
2. Whiffin N, Minikel E, Walsh R, O'Donnell-Luria AH, Karczewski K, Ing AY, Barton PJR, Funke B, Cook SA, Macarthur D, et al. Using high-resolution variant frequencies to empower clinical genome interpretation. *Genet Med*. 2017;19(10):1151–1158.
3. Compeau PEC, Pevzner PA, Tesler G, Papoutsoglou G, Roscito JG, Dahl A, Myers G, Winkler S, Pippel M, Sameith K, et al. Cutadapt removes adapter sequences from high-throughput sequencing reads. *EMBnet J*. 2013;17(1):10–12.
4. Bolger AM, Lohse M, Usadel B. Trimmomatic: A flexible trimmer for Illumina sequence data. *Bioinformatics*. 2014;30(15):2114–2120.
5. Li H. Aligning sequence reads, clone sequences and assembly contigs with BWA-MEM. 2013.
6. DePristo MA, Banks E, Poplin R, Garimella K V, Maguire JR, Hartl C, Philippakis AA, del Angel G, Rivas MA, Hanna M, et al. A framework for variation discovery and genotyping using next-generation DNA sequencing data. *Nat Genet*. 2011;43.
7. Broad Institute. Picard Toolkit. 2019.
8. Richards S, Aziz N, Bale S, Bick D, Das S, Gastier-Foster J, Grody WW, Hegde M, Lyon E, Spector E, et al. Standards and guidelines for the interpretation of sequence variants: A joint consensus recommendation of the American College of Medical Genetics and Genomics and the Association for Molecular Pathology. *Genet Med*. 2015;17(5):405–424.
9. Kramer CM, Appelbaum E, Desai MY, Desvigne-Nickens P, DiMarco JP, Friedrich MG, Geller N, Heckler S, Ho CY, Jerosch-Herold M, et al. Hypertrophic Cardiomyopathy Registry: The rationale and design of an international, observational study of hypertrophic cardiomyopathy. *Am Heart J*. 2015;170(2):223–230.
10. Abraham G, Qiu Y, Inouye M. FlashPCA2: principal component analysis of Biobank-scale genotype datasets. *Bioinformatics*. 2017;33(17):2776–2778.
11. Auton A, Brooks LD, Durbin RM, Garrison EP, Kang HM, Korbel JO, Marchini JL, McCarthy S, McVean GA, Abecasis GR. A global reference for human genetic variation. *Nature*. 2015;526(7571):68–74.
12. Lek M, Karczewski KJ, Minikel E V., Samocha KE, Banks E, Fennell T, O'Donnell-Luria AH, Ware JS, Hill AJ, Cummings BB, et al. Analysis of protein-coding genetic variation in 60,706 humans. *Nature*. 2016;536(7616):285–291.
13. Karczewski KJ, Francioli LC, Tiao G, Cummings BB, Alföldi J, Wang Q, Collins RL, Laricchia KM, Ganna A, Birnbaum DP, et al. Variation across 141,456 human exomes and genomes reveals the spectrum of loss-of-function intolerance across human protein-coding genes. *bioRxiv*. 2019:531210.
14. NHLBI Trans-Omics for Precision Medicine (TOPMed) Whole Genome Sequencing Program. BRAVO variant browser. 2018.
15. Taliun D, Harris D, Kessler MD, Carlson JZ, Szpiech Z, Torres R, Taliun SAG, Corvelo A, Stephanie M, Albert C, et al. Sequencing of 53 , 831 diverse genomes from the NHLBI TOPMed Program. *bioRxiv*. 2019:1–46.
16. NIHR BioResource. Whole-genome sequencing of rare disease patients in a national healthcare system. *bioRxiv*. 2019:507244.

17. Forster JJ, Smith PWF. for Binomial and Multinomial Logistic Regression Models. *Stat Sci*. 2003;(1989):169–177.
