# Supplementary material for: Reevaluation of the South Asian MYBPC3Δ25bp Intronic Deletion in Hypertrophic Cardiomyopathy
Source: Circ Genom Precis Med. 2020 Mar 12;13(3):e002783. doi: 10.1161/CIRCGEN.119.002783 (PMC7299222; doi:10.1161/CIRCGEN.119.002783)
Supplement: Supplementary file 2 [file hcg-13-e002783-s002.pdf]

A Re-evaluation of the South Asian *MYBPC3*$^{\Delta 25bp}$ Intronic Deletion in Hypertrophic Cardiomyopathy

Running title: *Harper et al.; South Asian MYBPC3*$^{\Delta 25}$ *Intronic Deletion in HCM*

Andrew R. Harper, MSc, MRCP[1,2]; Michael Bowman, BSc[3]; Jesse B.G. Hayesmoore, PhD[3]; Helen Sage, MSc[3]; Silvia Salatino, PhD[2]; Edward Blair, BMSc, FRCP[4]; Carolyn Campbell, FRCPath[3]; Bethany Currie, BSc[3]; Anuj Goel, MBBS, MSc[1,2]; Karen McGuire, BSc[3]; Elizabeth Ormondroyd, PhD[1,5]; Kate Sergeant, PhD[3]; Adam Waring, BSc[2]; Jessica Woodley, FRCPath[6]; Christopher M. Kramer, MD[7]; Stefan Neubauer, MD[1]; HCMR Investigators; Martin Farrall, FRCPath[1,2]; Hugh Watkins, MD, PhD[1,2]*, Kate L. Thomson, FRCPath PhD[1-3]*

[1]Radcliffe Department of Medicine, University of Oxford, Division of Cardiovascular Medicine, John Radcliffe Hospital; [2]Wellcome Centre for Human Genetics; [3]Oxford Medical Genetics Laboratories, Churchill Hospital; [4]Oxford Centre for Genomic Medicine, Oxford University Hospitals NHS Foundation Trust; [5]Division of Cardiovascular Medicine, John Radcliffe Hospital, Oxford; [6]West Midlands Regional Genetics Laboratory, Birmingham Woman's and Children's NHS Foundation Trust, Birmingham, United Kingdom; [7]University of Virginia Health System, Charlottesville, VA

*contributed equally

Circulation: Genomic and Precision Medicine

Correspondence:

Dr Kate Thomson FRCPath DPhil

Principal Clinical Scientist for Cardiac Genetics

Oxford Medical Genetics Laboratory

Oxford University Hospitals NHS Foundation Trust

The Churchill Hospital

Oxford, OX3 7LE, United Kingdom

Tel: +44(0)1865 225591

Email: Kate.Thomson@ouh.nhs.uk

Journal Subject Terms: Genetics; Etiology; Diagnostic Testing; Cardiomyopathy

Abstract

Background - The common intronic deletion, *MYBPC3*$^{\Delta25}$, detected in 4-8% of South Asian populations, is reported to be associated with cardiomyopathy, with ~7-fold increased risk of disease in variant carriers. Here we examine the contribution of *MYBPC3*$^{\Delta25}$ to hypertrophic cardiomyopathy (HCM) in a large patient cohort.

Methods - Sequence data from two HCM cohorts (n=5,393) was analysed to determine *MYBPC3*$^{\Delta25}$ frequency and co-occurrence of pathogenic variants in HCM genes. Case-control and haplotype analyses were performed to compare variant frequencies and assess disease association. Analyses were also undertaken to investigate the pathogenicity of a candidate variant, *MYBPC3* c.1224-52G>A.

Results - Our data suggest that the risk of HCM, previously attributed to *MYBPC3*$^{\Delta25}$, can be explained by enrichment of a derived haplotype, *MYBPC3*$^{\Delta25/-52}$, whereby a small subset of individuals bear both *MYBPC3*$^{\Delta25}$ and a rare pathogenic variant, *MYBPC3* c.1224-52G>A. The intronic *MYBPC3* c.1224-52G>A variant, which is not routinely evaluated by gene panel or exome sequencing, was detected in ~1% of our HCM cohort.

Conclusions - The *MYBPC3* c.1224-52G>A variant explains the disease risk previously associated with *MYBPC3*$^{\Delta25}$ in the South Asian population and is one of the most frequent pathogenic variants in HCM in all populations; genotyping services should ensure coverage of this deep intronic mutation. Individuals carrying *MYBPC3*$^{\Delta25}$ alone are not at increased risk of HCM and this variant should not be tested in isolation; this is important for the large majority of the 100 million individuals of South Asian ancestry who carry *MYBPC3*$^{\Delta25}$ and would previously have been declared at increased risk of HCM.

American Heart Association.
Circulation: Genomic and Precision Medicine

Key words: hypertrophic cardiomyopathy; genetic testing; variation; ethnicity; genetic association; South Asian population

Nonstandard Abbreviations and Acronyms

**ACTC1**	Actin Alpha Cardiac Muscle 1
**BRRD**	NIHR Bioresource for Rare Disease
**BWA**	Burrows-Wheeler Aligner
**GATK**	Genomic Analysis Toolkit
**HCM**	Hypertrophic cardiomyopathy
**HCMR**	Hypertrophic Cardiomyopathy Registry
**MYBPC3**	Myosin Binding Protein C
**MYH7**	Myosin Heavy Chain 7
**TNNI3**	Troponin I 3
**TNNT2**	Troponin T 2
**MYL2**	Myosin Light Chain 2
**MYL3**	Myosin Light Chain 3
**OMGL**	Oxford Medical Genetics Laboratory
**TPM1**	Tropomyosin 1

Circulation: Genomic and Precision Medicine

Introduction

Hypertrophic cardiomyopathy (HCM) is the most common inherited cardiac condition, affecting at least ~1:500 individuals.[1] It is a genetically heterogeneous disorder, typically attributable to pathogenic variants in genes encoding cardiac sarcomere proteins, predominantly *MYBPC3* and *MYH7*.[2] Truncating variants in *MYBPC3* are a well-recognized cause of HCM and the majority are considered to cause autosomal dominant disease with high age-related penetrance; consequently, such variants are extremely rare in the wider non-disease population.[2]

A 25 base pair deletion located within intron 32 of *MYBPC3* (*MYBPC3*$^{\Delta25}$), the c.3628-41_3628-17del variant, is a notable exception. Detected in 4-8% of individuals of South Asian ancestry,[3,4] and with an estimated 100 million carriers worldwide, this common variant is considered to be associated with cardiomyopathy, with an almost 7-fold increased risk of cardiomyopathy in heterozygous carriers.[3] Although previous studies have considered the possibility that *MYBPC3*$^{\Delta25}$ lies in linkage disequilibrium with another *MYBPC3* variant that causes or contributes to disease risk,[3,4] comprehensive analyses in large patient cohorts have not been performed.

Circulation: Genomic and Precision Medicine

Here, using genetic data from two large HCM cohorts, we present data suggesting that *MYBPC3*$^{\Delta25}$ is not a pathogenic risk factor in HCM. Rather, the increased frequency of this variant in South Asian cardiomyopathy cohorts reflects the enrichment of a derived haplotype, which bears both the common *MYBPC3*$^{\Delta25}$ variant and a rare pathogenic variant, *MYBPC3* c.1224-52G>A. Additionally, we find that *MYBPC3* c.1224-52G>A, an intronic variant which is not routinely detected on gene panel or exome sequencing, is the single most common pathogenic variant in individuals of South Asian ancestry in our cohort, and the second most common in individuals of European ancestry.

Methods

The complete methods are available in the Supplemental Material section. Due to the confidential nature of some of the research materials supporting this publication not all of the data can be made accessible to other researchers. Please contact the corresponding author for more information. The study was approved by the local ethics committees and all patients signed an informed consent.

Results

OMGL demographic and clinical details

Within the OMGL cohort, demographic information was available for 98.0% of individuals (2,703/2,757). The majority of referrals were provided by inherited cardiac condition centres within the United Kingdom (80.1%, 2,166/2,757). The average age was 54.5 years (±16.2) and 68.4% were male (n=1,845) (Table 1). No self-identified, or genetically derived, ancestry information was available.

American Heart Association.
Circulation: Genomic and Precision Medicine

HCMR demographic and clinical details

Within the HCMR cohort, the average age was 49.5 years (± 11.3) and 71.4% were male. Genetically-derived ancestry predictions, determined through principal components analysis, demonstrated European ancestry in 78.3%, African ancestry in 9.0% and South Asian ancestry in 5.1% of individuals (Table 1).

Population frequency of *MYBPC3*$^{\Delta 25}$

In the Genome Aggregation Database (gnomAD v2.1.1), 6.2% of individuals ascribed South Asian ancestry were heterozygous for the *MYBPC3*$^{\Delta 25}$ variant (943/15,296, [95% CI 5.7-6.5%]), 0.1% were homozygous (19). This is consistent with previous studies which have reported

frequencies ranging from 2 to 8%.[3,4] The *MYBPC3*$^{\Delta25}$ variant is highly specific to individuals of South Asian ancestry: 98.1% [95% CI: 97.0–98.9%] of *MYBPC3*$^{\Delta25}$ variant carriers within gnomAD are derived from a South Asian population (Table 2).

Oxford clinical laboratory cohort

In the OMGL HCM cohort, pathogenic variants were detected in 17.1% (471/2,757), likely pathogenic variants in 6.9% (191/2,757), and variants of uncertain significance in an additional 14.2% (392/2,757) of individuals. A summary of the most frequently detected variants is presented in Supplementary Table 1. 0.7% (20/2,757) of individuals were heterozygous for the *MYBPC3*$^{\Delta25}$ variant. In 50.0% (10/20) of individuals heterozygous for *MYBPC3*$^{\Delta25}$, a pathogenic or likely pathogenic sarcomeric gene variant was also detected; variants of uncertain clinical significance were detected in an additional three individuals (15.0%, 3/20 (Table 3). Of these accompanying variants, *MYBPC3* c.1224-52G>A was the most frequently observed, found in 30.0% (6/20) of individuals heterozygous for *MYBPC3*$^{\Delta25}$.

American Heart Association.
Circulation: Genomic and Precision Medicine

HCMR cohort

In the HCMR cohort, pathogenic variants were detected in 21.7% (572/2,636), likely pathogenic variants in 8.2% (216/2,636) and variants of uncertain significance in an additional 14.4% (379/2,636) of individuals. A summary of the most frequently detected variants is presented in Supplementary Table 1. 0.7% of individuals (18/2,636) were heterozygous for the *MYBPC3*$^{\Delta25}$ variant, no homozygous individuals were detected; 17 *MYBPC3*$^{\Delta25}$ variant carriers were ascribed as South Asian ancestry by genetic principal components analysis (94.4%, 17/18). The carrier frequency for *MYBPC3*$^{\Delta25}$ within the HCMR South Asian ancestry group was 12.7% [95% CI 8.1-19.4%] (17/134).

In 58.8% (10/17) of South Asian individuals heterozygous for *MYBPC3*$^{\Delta25}$, a pathogenic

variant in one of the sarcomeric genes was detected (Table 3). An additional two individuals were found to have variants of uncertain clinical significance (11.8%, 2/17). Replicating findings from our discovery cohort, the c.1224-52G>A variant was the most frequent, found in 29.4% (5/17) of South Asian individuals heterozygous for *MYBPC3*$^{\Delta25}$.

Overall, including the *MYBPC3* c.1224-52G>A variant, 25.4% [95% CI: 18.8 – 33.4] (34/134) of HCMR probands ascribed South Asian ancestry had a pathogenic or likely pathogenic sarcomeric gene variant. An additional 15.6% [95% CI: 10.5 – 22.8] (21/134) harboured a variant of uncertain significance. This is comparable to the detection rate in the OMGL cohort, and to previously published cohorts.[2,5]

Direct comparison of the proportion of heterozygous *MYBPC3*$^{\Delta25}$ variant carriers between the HCMR (17/134) and gnomAD (943/15,296) South Asian cohorts indicated a 2-fold enrichment within HCM cases, (OR: 2.1 [95% CI 1.2-3.4], p-value=0.008). When HCMR probands with the *MYBPC3*$^{\Delta25/-52}$ haplotype were excluded, no difference was observed (OR: 0.96 [95% CI 0.40-1.95], p-value=1.0). Exact multivariate logistic regression, of individuals of South Asian ancestry from the HCMR and BRRD cohorts (Table 4), provided evidence in support of disease association for the *MYBPC3* c.1224-52G>A variant (OR: 15.90; 95% CI: 2.05 – ∞; p-value=0.003), but not the *MYBPC3*$^{\Delta25}$ variant (OR 1.76; 95% CI: 0.77 – 4.36; p-value=0.15). The significance of the *MYBPC3* c.1224-52G>A association adjusted for the *MYBPC3*$^{\Delta25}$ variant was confirmed using an exact Mantel-Haenszel test (p-value 0.003).

American Heart Association.
Circulation: Genomic and Precision Medicine

In individuals of South Asian ancestry in the HCMR cohort, the *MYBPC3* c.1224-52G>A variant was found to occur on the second most commonly observed *MYBPC3*$^{\Delta25}$ haplotype (Figure 1). Hence, there is evidence of strong linkage disequilibrium between *MYBPC3*$^{\Delta25}$ and *MYBPC3* c.1224-52G>A (D′ = 0.81 and r^2 = 0.22) (Supplementary Figure 1 and Supplementary

Table 2). In South Asian individuals, the *MYBPC3* c.1224-52G>A variant also occurred on a haplotype that did not include the *MYBPC3*$^{\Delta 25}$ variant.

Investigating the pathogenicity of *MYBPC3* c.1224-52G>A

The *MYBPC3* c.1224-52G>A variant (Chr11(GRCh37):g.47364865C>T, NM_000256.3) was detected in 32 of 2,757 (1.2% [95% CI: 0.8-1.6%]) probands in the OMGL cohort and in 23 of 2,636 (0.9%, [95% CI: 0.6-1.2%]) probands in the HCMR cohort. A two-sample test for equality of proportions, with continuity correction, suggests the minor allele frequencies derived from OMGL and HCMR are equivalent (p-value = 0.98). No other pathogenic or likely pathogenic sarcomere gene variants were detected in these cases. Within the OMGL cohort, *MYBPC3* c.1224-52G>A was confirmed to co-segregate with HCM in four families (Supplementary Figure 2); in three it was detected in the proband and two other affected relatives. Within the wider HCMR and OMGL populations, *MYBPC3* c.1224-52G>A was found to occur on two additional haplotypes, distinct from the two South Asian haplotypes, which argues against a unique founder mutation.

American Heart Association.
Circulation: Genomic and Precision Medicine

The c.1224-52G>A variant occurs once within 76,048 non-overlapping individuals, present within gnomAD (v.2.1.1) and NHLBI TOPMed (https://bravo.sph.umich.edu/freeze5/hg38/), indicating a global minor allele frequency, incorporating all available ancestral groups, of 6.57 x 10^{-6}. A comparison of the proportion of individuals heterozygous for this variant in the combined OMGL and HCMR cohorts (55/5,393), against these reference populations, generates an extreme effect size (OR: 780, [95% CI: 135-16,384]; p-value=9.74 x 10^{-64}).

In silico splice site tools predict that c.1224-52G>A introduces a cryptic splice acceptor site in intron 13 (NM_000256.3), 50 nucleotides upstream (5') of the native site. PCR of cDNA

reverse transcribed from RNA from two individuals with the c.1224-52G>A variant generated an aberrant product. Sequencing of this product confirmed *in silico* predictions and showed inclusion of 50 intronic nucleotides in the transcript (Figure 2). Inclusion of these nucleotides is predicted to lead to a frameshift in the amino acid sequence and insertion of a premature termination codon at position 438 (p.Ser408fs*31).

Pathogenicity classification for *MYBPC3* c.1224-52G>A

Using the American College of Medical Genetics (ACMG) framework[6], the *MYBPC3* c.1224-52G>A variant was classified as pathogenic based on the following criteria: PS3: RNA studies have provided evidence of an aberrant effect on splicing (our analyses and published data[7]); PS4: The variant is significantly more frequent in probands with HCM than in population controls; PM2: The variant is very rare in the wider population; and PP1: There is evidence of co-segregation with HCM in multiple families (four in our cohort and published data[7]).

Circulation: Genomic and Precision Medicine

Discussion

When the *MYBPC3*$^{\Delta 25}$ variant was first reported to be associated with cardiomyopathy in the South Asian population it was thought likely to have a direct role in disease pathogenesis; since the initial report, it has come to be considered as one of the most compelling examples of a common, low-penetrance variant contributing to the genetic architecture of HCM.[3,8–12] Genetic analyses undertaken in this study challenge these previous assertions and show that the *MYBPC3*$^{\Delta 25}$ variant does not directly confer an increased risk of cardiomyopathy, but instead acts as a proxy marker for a rare, large effect size, intronic pathogenic variant, *MYBPC3* c.1224-52G>A (Figure 3). Consequently, we conclude that heterozygosity for the *MYBPC3*$^{\Delta 25}$ common variant is not pathogenic for HCM.

Through RNA studies and segregation analyses we provide robust evidence to support the pathogenicity of the *MYBPC3* c.1224-52G>A variant. This variant has previously been described in the literature as a pathogenic variant[7] however, neither its high prevalence, nor its relationship with *MYBPC3*$^{\Delta25}$, has been reported. Our analyses reveal *MYBPC3* c.1224-52G>A to be a recurrent variant, and one of the most frequent pathogenic variants across all known HCM genes in both European and South Asian populations, comparable to other well-established recurrent and founder pathogenic variants (e.g. *MYBPC3* c.2373dup[13] and *MYBPC3* p.Glu258Lys[2]), and exceeded only by the *MYBPC3* p.Arg502Trp variant, the most common pathogenic variant in HCM[2,5,14]. Further, the *MYBPC3* c.1224-52G>A variant has a strikingly high odds ratio for disease (~700), suggesting that it is a high penetrance allele.

Haplotype analyses indicate that an ancestral *MYBPC3* c.1224-52G>A variant arose on a haplotype bearing the common *MYBPC3*$^{\Delta25}$ variant, and that the reported association between *MYBPC3*$^{\Delta25}$ and HCM in the South Asian population was due to the increased frequency of the derived *MYBPC3*$^{\Delta25/-52}$ haplotype, which had not previously been differentiated from the common *MYBPC3*$^{\Delta25}$ haplotype. In our cohort, after accounting for the *MYBPC3*$^{\Delta25/-52}$ haplotype, the frequency of the *MYBPC3*$^{\Delta25}$ allele appears equivalent between HCM cases and reference controls, which casts doubt upon previous pathogenic inferences from risk-associations and suggests that it is not clinically appropriate to type the *MYBPC3*$^{\Delta25}$ in isolation. Indeed, the ability to detect the *MYBPC3*$^{\Delta25/-52}$ haplotype is critical not only for individuals with a clinical diagnosis of HCM, but for the vast majority of the 100 million individuals of South Asian ancestry heterozygous for the *MYBPC3*$^{\Delta25}$ alone, who would previously have been declared at increased risk of HCM.

American Heart Association.
Circulation: Genomic and Precision Medicine

Limitations

Our conclusions rely on the observed *MYBPC3*$^{\Delta 25}$ and *MYBPC3*$^{\Delta 25/\text{-}52}$ haplotype frequencies being representative of the wider South Asian population. Here, direct evaluation of *MYBPC3*$^{\Delta 25}$ and *MYBPC3*$^{\Delta 25/\text{-}52}$ and HCM disease risk has relied on analysis performed using individuals ascribed South Asian ancestry based on genetic principal components analysis from two independent, but relatively small, cohorts. Large reference cohorts, specifically gnomAD and TOPMed, were useful in quantifying the allele frequencies of both *MYBPC3*$^{\Delta 25}$ and *MYBPC3* c.1224-52G>A, but were not suitable for the direct evaluation of the *MYBPC3*$^{\Delta 25/\text{-}52}$ haplotype, given the lack of individual-level data.

Our case series comprised two large HCM cohorts with a combined total of 5,394 HCM probands (OMGL n=2,757 and HCMR n=2,636), representing the largest published HCM cohort to date. *MYBPC3*$^{\Delta 25}$ and *MYBPC3*$^{\Delta 25/\text{-}52}$ haplotype frequencies were equivalent within these mixed ancestry HCM cohorts. Ancestry data were only available from the HCMR cohort, in which 134 cases were defined as South Asian; additional analyses in other South Asian cohorts will refine *MYBPC3*$^{\Delta 25/\text{-}52}$ haplotype frequency estimates, and allow more accurate quantification of the strength of the association of this haplotype to HCM in this population.

American Heart Association.
Circulation: Genomic and Precision Medicine

The findings in this study relate specifically to HCM. In the original case-control study by Dhandapany *et al*, two composite case groups were assembled that included individuals diagnosed with HCM (n=357), dilated cardiomyopathy (DCM) (n=395), and restrictive cardiomyopathy (RCM) (n=15)[3]. Whilst our findings refute a pathogenic role for the *MYBPC3*$^{\Delta 25}$ variant in HCM, at present, our conclusions do not extend to these other cardiomyopathies, or to homozygosity for this variant. However, given current understanding of the diametrically opposing molecular mechanisms that underpin sarcomeric HCM and DCM[15–17], it seems unlikely

that a single variant, such as *MYBPC3*$^{\Delta 25}$, could cause both conditions. Further, truncating variants in *MYBPC3* have only been associated with HCM, and not primary DCM.[2]

Conclusions

The results of this study provide strong evidence to refute a direct pathogenic link between the *MYBPC3*$^{\Delta 25}$ variant and HCM risk; this is important for the very large number of South Asian individuals who will be found to have this variant when undergoing either targeted or genome-wide genetic analysis. Additionally, they highlight *MYBPC3* c.1224-52G>A as an important HCM variant. They also reiterate the importance of sequencing deeper intronic regions in the *MYBPC3* gene, and, indeed, other cardiomyopathy genes where truncating variants are believed to cause the disease. Collectively, these findings have significant implications for our understanding of the genetic architecture of HCM and for the clinical management of patients with HCM.

American Heart Association.
Circulation: Genomic and Precision Medicine

Acknowledgments: We thank the NIHR BioResource volunteers for their participation, and gratefully acknowledge NIHR BioResource centres, NHS Trusts and staff for their contribution. We thank the National Institute for Health Research and NHS Blood and Transplant. The views expressed are those of the author(s) and not necessarily those of the NHS, the NIHR or the Department of Health and Social Care.

Sources of Funding: ARH has received support from the Medical Research Council Doctoral Training Partnership. AG has received support from the British Heart Foundation, European Commission [LSHM-CT- 2007-037273, HEALTH-F2-2013-601456] and TriPartite Immunometabolism Consortium [TrIC]- NovoNordisk Foundation [NNF15CC0018486]. AW has received support from the Wellcome Trust. MF and HW have received support from the Wellcome Trust core award [090532/Z/09/Z] and the BHF Centre of Research Excellence, Oxford [RE/13/1/30181]. HW has received support from the National Institute for Health Research (NIHR) Oxford Biomedical Research Centre. CK, SN and HW received support from a

National Heart, Lung, and Blood Institute [grant U01HL117006-01A1]. Computation used the Oxford Biomedical Research Computing facility, a joint development between the Wellcome Centre for Human Genetics and the Big Data Institute supported by Health Data Research UK and the NIHR Oxford Biomedical Research Centre. Financial support was provided by the Wellcome Trust Core Award [Grant Number 203141/Z/16/Z].

Appendix: HCMR Investigators

Theodore Abraham, MD - Hypertrophic Cardiomyopathy Ctr of Excellence, Johns Hopkins Univ, Baltimore, MD
Lisa Anderson, MD - St Georges Univ Hospitals NHS Trust, London, United Kingdom
Evan Appelbaum, MD - Depts of Medicine, Cardiovascular Division & Radiology, Beth Israel Deaconess Medical Ctr, Harvard Medical School, Boston, MA
Camillo Autore, MD - Division of Cardiology, Dept of Clinical & Molecular Medicine, St. Andrea Hospital, Sapienza Univ, Rome, Italy
Colin Berry, MD - British Heart Foundation Glasgow Cardiovascular Research Ctr, Inst of Cardiovascular & Medical Sciences, Univ of Glasgow, UK
Elena Biagini, MD - Cardio-Thoraco-Vascular Dept, Univ Hospital of Bologna, Policlinico S. Orsola-Malpighi, Bologna, Italy
William Bradlow, MD - Dept of Cardiology, New Queen Elizabeth Hospital Birmingham, UK
Chiara Bucciarelli-Ducci, MD - Bristol Heart Inst, Bristol National Inst of Health Research (NIHR) Biomedical Research Ctr, Univ Hospitals Bristol NHS Trust & Univ of Bristol, UK
Amedeo Chiribiri, MD, PhD - Cardiovascular Division, King's College London British Heart Foundation Ctr of Excellence, The Rayne Inst, St. Thomas' Hospital Campus, London, UK
Lubna Choudhury, MD - Division of Cardiology, Dept of Medicine, Bluhm Cardiovascular Inst, Northwestern Univ Feinberg School of Medicine, Chicago, IL
Andrew Crean, MD - Division of Cardiology, Peter Munk Cardiac Center, Univ Health Network, Univ of Toronto, Ontario, Canada
Dana Dawson, MD - Aberdeen Cardiovascular & Diabetes Ctr, Univ of Aberdeen, UK
Milind Y. Desai, MD - Dept of Cardiovascular Medicine, Center for Radiation Heart Disease, Heart & Vascular Inst, Cleveland Clinic, Cleveland, OH
Eleanor Elstein, MD - Division of Cardiology, Dept of Medicine, Royal Victoria Hospital, McGill Univ Health Ctr, Montreal, Quebec, Canada
Andrew Flett, MD - Dept of Cardiology, Univ Hospital Southampton NHS Foundation Trust, Southampton, UK
Matthias Friedrich, MD - Dept of Medicine, Heidelberg Univ, Heidelberg, Germany
Stephen Heitner, MD - Oregon Health & Sciences Univ (OHSU), Division of Cardiovascular Medicine, Knight Cardiovascular Institute, Portland, OR
Adam Helms, MD - Dept of Internal Medicine, Univ of Michigan, Ann Arbor, MI
Carolyn Ho, MD - Cardiovascular Division, Brigham and Women's Hospital, Boston, MA
Daniel L. Jacoby, MD - Section of Cardiovascular Medicine, Dept of Internal Medicine, Yale School of Medicine, New Haven, CT
Han Kim, MD - Duke Cardiovascular Magnetic Resonance Center & Division of Cardiology, Duke Univ Medical Center, Durham, NC
Bette Kim, MD - Mount Sinai West, Icahn School of Medicine at Mount Sinai, New York City, NY
Eric Larose, MD - Quebec Heart & Lung Inst, Laval Univ, Quebec, Canada
Masliza Mahmod, MD - Division of Cardiovascular Medicine, Radcliffe Dept of Medicine, Univ of Oxford, UK

Circulation: Genomic and Precision Medicine

Heiko Mahrholdt, MD - Dept of Cardiology, Robert-Bosch-Krankenhaus, Stuttgart, Germany
Martin Maron, MD - Hypertrophic Cardiomyopathy Center & Research Institute, Tufts Medical Ctr, Boston, MA
Gerry McCann, MD - Dept of Cardiovascular Sciences, Univ of Leicester, UK
Michelle Michaels, MD - Erasmus Univ, Rotterdam, the Netherlands
Saidi Mohiddin, MD - Barts Heart Center, The Cardiovascular Magnetic Resonance Imaging Unit, St Bartholomew's Hospital, London, UK
Sherif Nagueh, MD - Methodist DeBakey Heart & Vascular Center, Houston, TX
David Newby, MD - Ctr for Cardiovascular Science, Univ of Edinburgh, UK
Iacopo Olivotto, MD - Cardiomyopathy Unit & Genetic Unit, Careggi Univ Hospital, Florence, Italy
Anjali Owens, MD - Ctr for Inherited Cardiovascular Disease, Division of Cardiovascular Medicine, Perelman School of Medicine, Univ of Pennsylvania, Philadelphia, PA
F. Pierre-Mongeon, MD - Montréal Heart Inst, Canada
Sanjay Prasad, MD - National Heart & Lung Inst, Imperial College London & Royal Brompton Hospital, London, UK
Ornella Rimoldi, MD - Vita Salute Univ & San Raffaele Hospital, Milan, Italy
Michael Salerno, MD - Dept of Medicine, Univ of Virginia, Charlottesville, VA
Jeanette Schulz-Menger, MD - Charité, Medical Faculty of the Humboldt Univ, Experimental & Clinical Research Center and Helios Clinics, Cardiology, Berlin, Germany
Mark Sherrid, MD - Hypertrophic Cardiomyopathy Program, Leon Charney Division of Cardiology, Dept of Medicine, New York Univ School of Medicine, New York, NY
Peter Swoboda, MD - Dept of Cardiovascular Imaging Science, Leeds Inst of Cardiovascular & Metabolic Medicine, Univ of Leeds, UK
Albert van Rossum, MD - Dept of Cardiology, Amsterdam UMC, HZ Amsterdam, the Netherlands
Jonathan Weinsaft, MD - Depts of Medicine & Radiology, Weill Cornell Medical College, New York, NY
James White, MD - Calgary Foothills Medical Ctr, Univ of Calgary, Alberta, Canada
Eric Williamson, MD - Dept of Radiology, Mayo Clinic, Rochester, MN

American Heart Association.
Circulation: Genomic and Precision Medicine

Disclosures: HW is a consultant for Cytokinetics. CK is a consultant for Cytokinetics and receives research grants from Biotelemetry and MyoKardia. SH is a consultant for Pfizer and Cytokinetics, and receives research grants from Boehringer Ingelheim. The other authors have no conflicts of interest to disclose.

References:

1. Maron BJ, Gardin JM, Flack JM, Gidding SS, Kurosaki TT, Bild DE. Prevalence of hypertrophic cardiomyopathy in a general population of young adults. Echocardiographic analysis of 4111 subjects in the CARDIA Study. Coronary Artery Risk Development in (Young) Adults. *Circulation*. 1995;92:785–9.

2. Walsh R, Thomson KL, Ware JS, Funke BH, Woodley J, McGuire KJ, Mazzarotto F, Blair E, Seller A, Taylor JC, et al. Reassessment of Mendelian gene pathogenicity using 7,855 cardiomyopathy cases and 60,706 reference samples. *Genet Med*. 2017;19:192–203.

3. Dhandapany PS, Sadayappan S, Xue Y, Powell GT, Rani DS, Nallari P, Rai TS, Khullar M,

Soares P, Bahl A, et al. A common MYBPC3 (cardiac myosin binding protein C) variant associated with cardiomyopathies in South Asia. *Nat Genet*. 2009;41:187–191.

4. Viswanathan SK, Puckelwartz MJ, Mehta A, Ramachandra CJA, Jagadeesan A, Fritsche-Danielson R, Bhat R V, Wong P, Kandoi S, Schwanekamp JA, et al. Association of Cardiomyopathy With MYBPC3 D389V and MYBPC3 Δ25bp Intronic Deletion in South Asian Descendants. *JAMA Cardiol*. 2018;3:481.

5. Alfares A a., Kelly M a., McDermott G, Funke BH, Lebo MS, Baxter SB, Shen J, McLaughlin HM, Clark EH, Babb LJ, et al. Results of clinical genetic testing of 2,912 probands with hypertrophic cardiomyopathy: expanded panels offer limited additional sensitivity. *Genet Med*. 2015;17:880–888.

6. Richards S, Aziz N, Bale S, Bick D, Das S, Gastier-Foster J, Grody WW, Hegde M, Lyon E, Spector E, et al. Standards and guidelines for the interpretation of sequence variants: A joint consensus recommendation of the American College of Medical Genetics and Genomics and the Association for Molecular Pathology. *Genet Med*. 2015;17:405–424.

7. Bagnall RD, Ingles J, Dinger ME, Cowley MJ, Ross SB, Minoche AE, Lal S, Turner C, Colley A, Rajagopalan S, et al. Whole Genome Sequencing Improves Outcomes of Genetic Testing in Patients With Hypertrophic Cardiomyopathy. *J Am Coll Cardiol*. 2018;72:419–429.

8. Bashyam MD, Purushotham G, Chaudhary AK, Rao KM, Acharya V, Mohammad TA, Nagarajaram HA, Hariram V, Narasimhan C. A low prevalence of MYH7/MYBPC3 mutations among Familial Hypertrophic Cardiomyopathy patients in India. *Mol Cell Biochem*. 2012;360:373–382.

9. Kuster DWD, Sadayappan S. MYBPC3's alternate ending: Consequences and therapeutic implications of a highly prevalent 25 bp deletion mutation. *Pflugers Arch*. 2014;466:207–213.

10. Srivastava A, Garg N, Mittal T, Khanna R, Gupta S, Seth PK, Mittal B. Association of 25 bp deletion in MYBPC3 gene with left ventricle dysfunction in coronary artery disease patients. *PLoS One*. 2011;6:1–7.

11. Kumar S, Mishra A, Srivastava A, Bhatt M, Garg N, Agarwal SK, Pande S, Mittal B. Role of common sarcomeric gene polymorphisms in genetic susceptibility to left ventricular dysfunction. *J Genet*. 2016;95:263–272.

12. Barefield DY, Lynch IV TL, Jagadeesan A. High-Throughput Diagnostic Assay for a Highly Prevalent Cardiomyopathy-Associated MYBPC3 Variant. *J Mol Biomark Diagn*. 2016;07.

13. Alders M, Jongbloed R, Deelen W, Van den Wijngaard A, Doevendans P, Cate F Ten, Regitz-Zagrosek V, Vosberg HP, Van Langen I, Wilde A, et al. The 2373insG mutation in the MYBPC3 gene is a founder mutation, which accounts for nearly one-fourth of the HCM cases in the Netherlands. *Eur Heart J*. 2003;24:1848–1853.

Circulation: Genomic and Precision Medicine

14. Saltzman AJ, Mancini-dinardo D, Li C, Chung WK, Carolyn Y, Hurst S, Wynn J, Care M, Hamilton RM, Gregor W, et al. The Cardiac Myosin Binding Protein C Arg502Trp Mutation: A Common Cause of Hypertrophic Cardiomyopathy. *Circ Res*. 2010;106:1549–1552.

15. Robinson P, Griffiths PJ, Watkins H, Redwood CS. Dilated and hypertrophic cardiomyopathy mutations in troponin and α-tropomyosin have opposing effects on the calcium affinity of cardiac thin filaments. *Circ Res*. 2007;101:1266–1273.

16. Debold EP, Schmitt JP, Patlak JB, Beck SE, Moore JR, Seidman JG, Seidman C, Warshaw DM. Hypertrophic and dilated cardiomyopathy mutations differentially affect the molecular force generation of mouse α-cardiac myosin in the laser trap assay. *Am J Physiol Heart Circ Physiol*. 2007;293:H284-91.

17. Yotti R, Seidman CE, Seidman JG. Advances in the Genetic Basis and Pathogenesis of Sarcomere Cardiomyopathies. *Annu Rev Genom Hum Genet*. 2019;20:129–153.

American Heart Association.

Circulation: Genomic and Precision Medicine

Table 1. Demographic summary for OMGL and HCMR cohorts.

	**OMGL**	**HCMR**
**Total (n)**	2,757	2,636
**Age (SD)**	54.5 (16.3)	49.5 (11.3)
**Male**	1,845 (68.4%)	1,893 (71.4%)
**Ancestry**		
AFR	NA	239 (9.0%)
AMR	NA	135 (5.1%)
EAS	NA	68 (2.6%)
EUR	NA	2,074 (78.3%)
SAS	NA	134 (5.1%)
**Variant carriers**	P: 471 (17.1%)	P: 572 (21.6%)
	LP: 191 (6.9%)	LP: 216 (8.2%)
	VUS: 392 (14.2%)	VUS: 379 (14.3%)
	Negative: 1,703 (61.8%)	Negative: 1,483 (56.0%)

SD = standard deviation. Ancestry codes as per the International Genome Sample Resource: AFR = African; AMR = Ad Mixed American; EAS = East Asian; EUR = European; SAS = South Asian. Counts for individuals with pathogenic (P), likely pathogenic (LP) or a variant of uncertain significance (VUS) included.

American Heart Association.
Circulation: Genomic and Precision Medicine

Table 2. Summary of allele frequency differences between cases and controls.

	Cases				Controls							
	**OMGL**	**HCMR**	**Total cases**	**P-value***	**BRRD**	**gnomAD exomes**	**gnomAD genomes**	**Total gnomAD**	**TOPMED**	**Total Controls†**	**OR (95% CI)**	**Fisher's P-value‡**
MYBPC3^{-52} minor allele frequency												
Global	0.00580 [0.00574 – 0.00587] (32/2,757)	0.00436 [0.00431 - 0.00442] (23/2,636)	0.00510 [0.00506 - 0.00514] (55/5,393)	0.359	- [0-3.25 x 10^{-7}] (0/6,056)	- (0/0)	3.2 x 10^{-5} [9.56 x 10^{-5} - 3.40 x 10^{-5}] (1/15,667)	3.2 x 10^{-5} [9.56 x 10^{-5} - 3.40 x 10^{-5}] (1/15,667)	- [0-3.13 x 10^{-8}] (0/62,784)	6.57 x 10^{-6} [1.97 x 10^{-5} - 7.00 x 10^{-6}] (1/76,048)	780 (135 – 16,384)	5.77 x 10^{-64}
European (NFE)	NA	0.00410 [0.00404 - 0.00416] (17/2,074)	0.00410 [0.00404 - 0.00416] (17/2,074)	-	- [0-5.45 x 10^{-7}] (0/3,606)	- (0/0)	- [0-2.55 x 10^{-7}] (0/7,696)	- [0-2.55 x 10^{-7}] (0/7,696)	No ancestry data	- [0-2.55 x 10^{-7}] (0/7,696)	∞ (15.4 – ∞)	3.43 x 10^{-12}
South Asian	NA	0.0224 [0.0218 – 0.0230] (6/134)	0.0224 [0.0218 – 0.0230] (6/134)	-	- [0-5.20 x 10^{-6}] (0/378)	- (0/0)	- (0/0)	- (0/0)	No ancestry data	- (0/0)	-	-
MYBPC3$^{\Delta 25}$ minor allele frequency												
Global	0.00363 [0.00358 - 0.00368] (20/2,757)	0.00341 [0.00336 - 0.00347] (18/2,636)	0.00352 [0.00349 - 0.00356] (38/5,393)	0.98	0.00182 [0.00179 - 0.00184] (22/6,056)	0.00394 [0.00393 - 0.00394] (978/124,259)	9.56 x 10^{-5} [9.22 x 10^{-5} - 9.91 x 10^{-5}] (3/15,695)	0.00350 [0.00350 - 0.00351] (981/139,954)	2.39 x 10^{-5} [2.30 x 10^{-5} - 2.48 x 10^{-5}] (3/62,784)	0.00250 [0.00249 - 0.00250] (984/197,114)	1.41 (0.99 – 1.96)	0.040
European (NFE)	NA	- [0-9.48 x 10^{-7}] (0/2,074)	- [0-9.48 x 10^{-7}] (0/2,074)	-	- [0-5.45 x 10^{-7}] (0/3,606)	8.90 x 10^{-6} [2.67 x 10^{-5} - 9.47 x 10^{-6}] (1/56,194)	- [0-2.55 x 10^{-7}] (0/7,708)	7.82 x 10^{-6} [2.34 x 10^{-5}- 8.33 x 10^{-6}] (1/63,902)	No ancestry data	7.82 x 10^{-6} [2.34 x 10^{-5}- 8.33 x 10^{-6}] (1/63,902)	-	-
South Asian	NA	0.0634 [0.0625 - 0.0644] (17/134)	0.0634 [0.0625 - 0.0644] (17/134)	-	0.0278 [0.0274 - 0.0282] (21/378)	0.0314 [0.0314 - 0.0315] (962/15,296)	- (0/0)	0.0321 [0.0320 - 0.0321] (981/15,296)	No ancestry data	0.0321 [0.0320 - 0.0321] (981/15,296)	1.98 (1.11 – 3.50)	0.015

Minor allele frequency [95% binomial confidence interval calculated using Wilson's method] presented with variant carrier counts in parentheses () beneath.
BRRD: BioResource for Rare Disease cohort; gnomAD: genome aggregation database; HCMR: Hypertrophic Cardiomyopathy Registry; NFE: Non-Finnish European; OMGL: Oxford Medical Genetics Laboratory; TOPMED: Trans-Omics for Precision Medicine.
*OMGL and HCMR case proportions compared using 2-sample test for equality of proportions with continuity correction.
†total controls calculated from non-overlapping samples provided by gnomAD and TOPMED.

‡ Fisher's P-value relates to the hypothesis that cases, derived from the OMGL and HCMR cohorts, are enriched for either *MYBPC3*$^{-52}$ or *MYBPC3*$^{\Delta 25}$ when compared with non-overlapping controls, provided by gnomAD and TOPMED.

Circulation: Genomic and Precision Medicine
American Heart Association.

Table 3. Pathogenic, likely pathogenic and variants of uncertain significance accompanying MYBPC3$^{\Delta 25}$ in individuals from both the OMGL and HCMR cohorts.

Gene	Variant	Variant classification	Frequency in individuals heterozygous for *MYBPC3*$^{\Delta 25bp}$
**OMGL**			
MYBPC3	c.1224-52G>A	Pathogenic	6/20
MYBPC3	c.1227-13G>A	Pathogenic	1/20
MYBPC3	c.2827C>T p.(Arg943Ter)	Pathogenic	1/20
MYH7	c.2770G>A p.(Glu924Lys)	Pathogenic	1/20
MYBPC3	c.2308G>A p.(Asp770Asn)	Likely pathogenic	1/20
MYBPC3	c.2030C>T p.(Pro677Leu)	VUS	1/20
MYH7	c.3931C>G p.(Gln1311Glu)	VUS	1/20
MYH7	c.436A>G p.(Lys146Glu)	VUS	1/20
**HCMR**			
MYBPC3	c.1224-52G>A	Pathogenic	5/18
MYBPC3	c.1227-13G>A	Pathogenic	1/18
MYBPC3	c.821+2T>C	Pathogenic	1/18
MYH7	c.1988G>A p.(Arg663His)	Pathogenic	1/18
MYH7	c.2221G>A p.(Gly741Arg)	Pathogenic	1/18
MYH7	c.5065C>T p.(Arg1689Cys)	VUS	1/18
MYH7	c.170G>A p.(Gly57Asp)	VUS	1/18

NCBI transcript IDs: *MYBPC3* NM_000256.3, *MYH7* NM_000257.2, NP_000248.2.

American Heart Association.
Circulation: Genomic and Precision Medicine

Table 4. South Asian cases vs controls.

	***MYBPC3*$^{\Delta25}$ carrier**		***MYBPC3*$^{\Delta25}$ non-carrier**	
	***MYBPC3* c.1224-52G>A carrier**	***MYBPC3* c.1224-52G>A non-carrier**	***MYBPC3* c.1224-52G>A carrier**	***MYBPC3* c.1224-52G>A non-carrier**
**Cases**	5	12	1	116
**Controls**	0	21	0	357

A 2-by-2-by-2 contingency table reporting counts of genotypes for cases vs. controls by indel carriers vs. non-carriers by -52 carriers vs. non-carriers for individuals of South Asian ancestry. Case data derived from HCMR and control data derived from BRRD.

American Heart Association.

Figure Legends:

Circulation: Genomic and Precision Medicine

Figure 1. Haplotype structure across *MYBPC3*. Each horizontal line (denoted A-G) represents a unique haplotype observed across *MYBPC3* with the South Asian population derived from the HCMR cohort (n=134). Figure generated from data provided by Haploview. Genetic markers denoted using the following nomenclature: <chromosome>_<GRCh37 position>_<reference allele>_<alternate allele>. Grey indicates presence of the ancestral allele. Blue shading indicates the presence of an alternate allele. The *MYBPC3*$^{\Delta25}$ allele (11_47353825_A_del25) is emphasised using a darker shade of blue. Red shading represents the presence of the *MYBPC3*$^{-52}$ allele (11_47364865_C_T). Haplotype A is composed entirely of reference alleles and is present in 49.9% of the cohort. *MYBPC3*$^{\Delta25}$ is present on haplotypes D and F. Haplotype F also includes *MYBPC3*$^{-52}$.

Figure 2. RNA studies *MYBPC3* c.1224-52G>A variant. Panel A: Gel-fractionation of RT-PCR products of lymphocyte-derived RNA from two affected individuals heterozygous for the *MYBPC3* c.1224-52A>G. Affected individuals in lanes 2 and 4 (corresponding reverse transcriptase negative controls in lanes 3 and 5) and controls in lanes 6, and 8 (corresponding reverse transcriptase negative controls in lanes 7 and 9). Blue arrow corresponds with normal fragment (323bp), as seen in controls, and the red arrow corresponds to the aberrant fragment (375bp). A 100 base pair ladder was used in lanes 1 and 11 (500bp [dense band], 400bp and 300bp bands shown). Panels B and C: Sanger sequencing of wild type (Panel B) and aberrant PCR product derived from cDNA of an affected individual harbouring MYBPC3 c.1224-52A>G (Panel C), indicates a 50 nucleotide intronic inclusion, confirming in silico splice site predictions.

American Heart Association.

Circulation: Genomic and Precision Medicine

Figure 3. A re-evaluation of the common *MYBPC3*Δ25bp intronic variant (*MYBPC3*$^{\Delta25}$) in the South Asian population. The *MYBPC3*$^{\Delta25}$ is a common variant present in 4-8% of the South Asian population (estimated to be carried by ~100 million people). In a cohort of South Asian HCM cases we detected a rare derived haplotype, bearing both *MYBPC3*$^{\Delta25}$ and a pathogenic variant, *MYBPC3* c.1224-52G>A. The rare *MYBPC3*$^{\Delta25/-52}$ haplotype is strongly associated with HCM with high penetrance. Haplotypes bearing *MYBPC3*$^{\Delta25}$ without the *MYBPC3* c.1224-52G>A variant, which account for the vast majority of South Asian individuals carrying the *MYBPC3*$^{\Delta25}$ variant, are not associated with HCM.

	11_47364865_C_T	11_47364832_C_T	11_47364709_C_T	11_47364377_T_C	11_47364163_G_A	11_47363535_C_T	11_47362774_G_A	11_47362758_C_T	11_47360235_G_A	11_47360129_C_T	11_47358997_G_A	11_47356671_G_A	11_47356628_G_C	11_47355473_C_T	11_47355234_G_A	11_47355104_G_A	11_47354857_C_T	11_47354847_G_A	11_47354787_C_T	11_47354196_A_C	11_47353825_A_del25	
A	C	C	C	T	G	C	G	C	G	C	G	G	G	C	G	G	C	G	C	A	A	49.9%
B	C	C	C	T	G	C	G	C	G	C	G	G	G	C	G	G	C	G	T	A	A	25.7%
C	C	C	C	T	G	C	G	C	G	C	A	G	G	C	G	G	C	G	T	A	A	6.3%
D	C	C	C	T	G	C	G	C	G	C	A	G	G	C	G	G	C	G	T	A	del25	2.9%
E	C	C	C	T	G	T	G	C	G	C	A	G	G	C	G	G	C	G	T	A	A	2.6%
F	T	C	C	T	G	C	G	C	G	C	A	G	G	C	G	G	C	G	T	A	del25	1.9%
G	C	C	C	T	G	C	G	C	G	C	G	A	G	C	G	G	C	G	C	A	A	1.2%

Circulation: Genomic and Precision Medicine

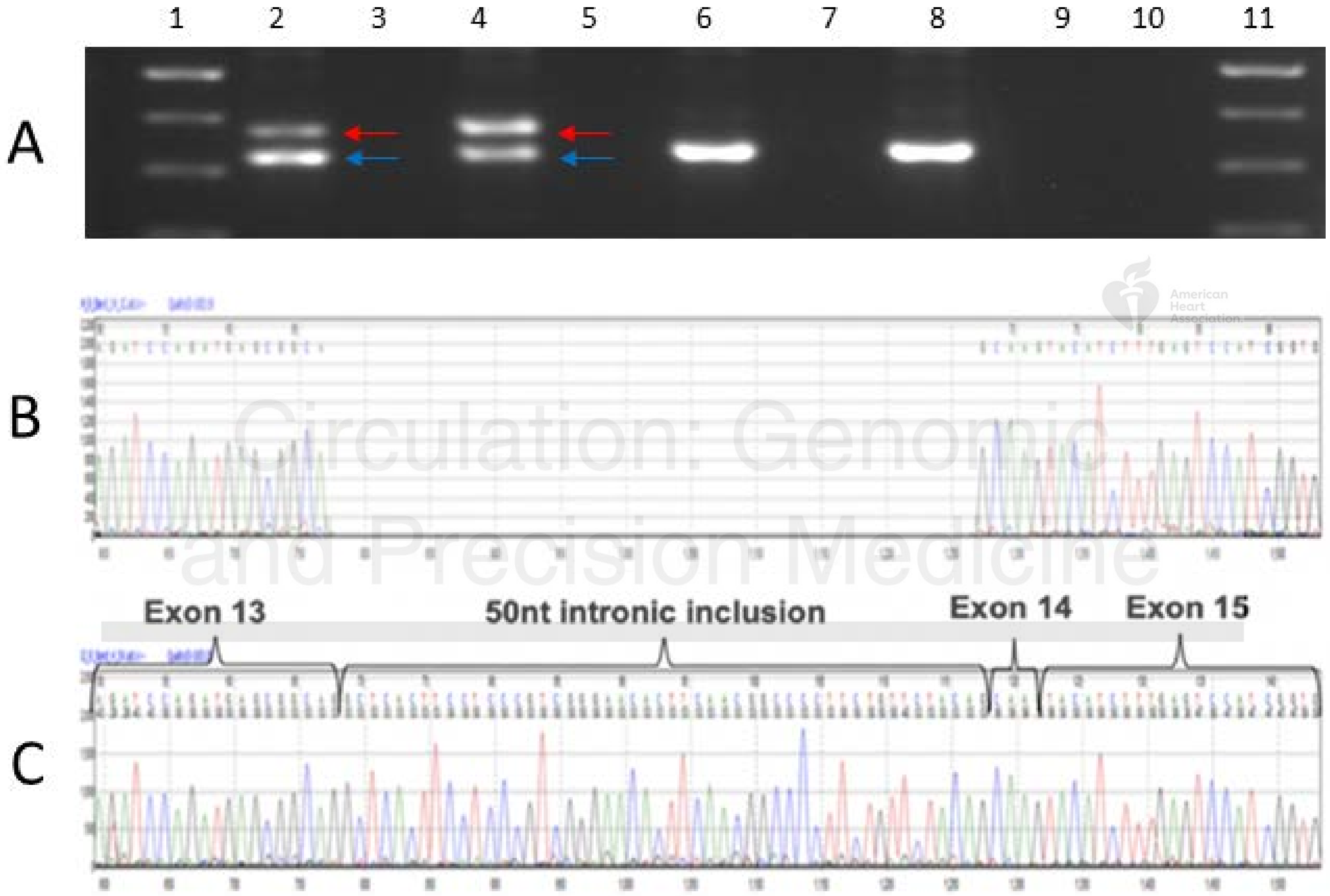

Circulation: Genomic and Precision Medicine

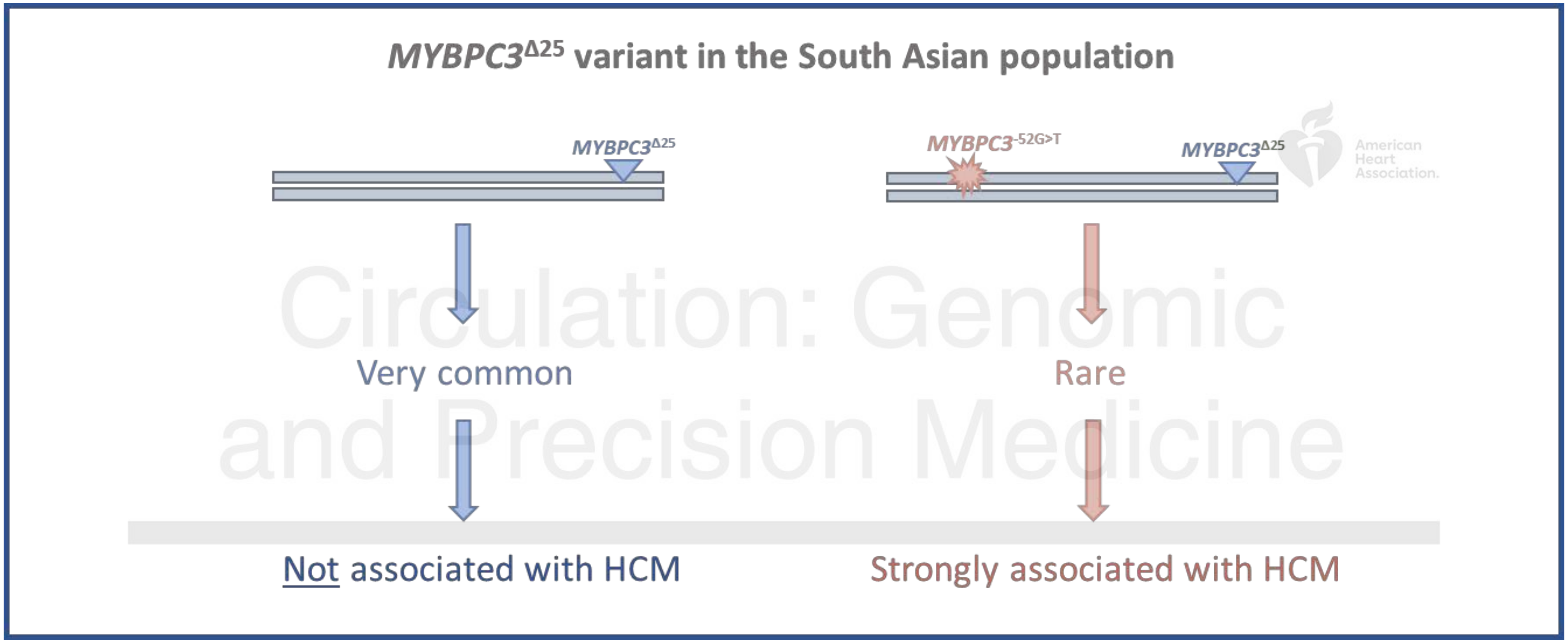

MYBPC3Δ25 variant in the South Asian population
MYBPC3Δ25
MYBPC3-52G>T
MYBPC3Δ25
Very common
Rare
Not associated with HCM
Strongly associated with HCM
American Heart Association.
Circulation: Genomic and Precision Medicine
